# Sequential changes in cellular properties accompanying amniote somite formation

**DOI:** 10.1111/joa.13791

**Published:** 2022-11-24

**Authors:** Agnieszka M. Piatkowska, Kaustubh Adhikari, Adam A. Moverley, Mark Turmaine, James A. Glazier, Nicolas Plachta, Susan E. Evans, Claudio D. Stern

**Affiliations:** ^1^ Department of Cell & Developmental Biology University College London, Gower Street (Anatomy Building) London UK; ^2^ Department of Intelligent Systems Engineering Biocomplexity Institute Bloomington Indiana USA; ^3^ Department of Cell and Developmental Biology, 9‐123 Smilow Center for Translational Research, Perelman School of Medicine University of Pennsylvania Philadelphia Pennsylvania USA; ^4^ Present address: The Open University Milton Keynes UK

**Keywords:** cell adhesion, cell polarity, epithelial–mesenchymal transition, mesenchymal–epithelial transition, segmentation, vertebral column

## Abstract

Somites are transient structures derived from the pre‐somitic mesoderm (PSM), involving mesenchyme‐to‐epithelial transition (MET) where the cells change their shape and polarize. Using Scanning electron microscopy (SEM), immunocytochemistry and confocal microscopy, we study the progression of these events along the tail‐to‐head axis of the embryo, which mirrors the progression of somitogenesis (younger cells located more caudally). SEM revealed that PSM epithelialization is a gradual process, which begins much earlier than previously thought, starting with the dorsalmost cells, then the medial ones, and then, simultaneously, the ventral and lateral cells, before a somite fully separates from the PSM. The core (internal) cells of the PSM and somites never epithelialize, which suggests that the core cells could be ‘trapped’ within the somitocoele after cells at the surfaces of the PSM undergo MET. Three‐dimensional imaging of the distribution of the cell polarity markers PKCζ, PAR3, ZO1, the Golgi marker GM130 and the apical marker N‐cadherin reveal that the pattern of polarization is distinctive for each marker and for each surface of the PSM, but the order of these events is not the same as the progression of cell elongation. These observations challenge some assumptions underlying existing models of somite formation.

## Graphical abstract

The process of epithelialization that culminates in the formation of somites begins very early in the pre‐somitic mesoderm, in defined order, starting dorsally, then medial and ventral, then lateral and finally the somite buds off – this challenges assumptions made by most models of somitogenesis.

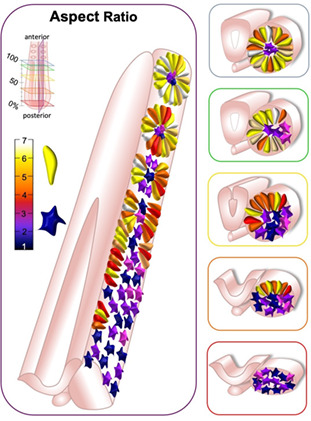

## 1 INTRODUCTION

Somites, first described by Marcello Malphigi (Malpighi, 1686), are transient structures, forming sequentially in head‐to‐tail order at regular time intervals. The pattern of somites is fundamental for the organization of the adult segmental body plan as it guides the associated pattern of peripheral nervous system elements (nerves, neural crest cells and ganglia) and generates the skeletal musculature as well as the vertebral column. In the last few decades, several models have been proposed to explain the mechanisms responsible for controlling the size, number and timing of somite formation in space and time (Piatkowska et al., 2021). These include pre‐patterning (Christ et al., 1974; Meier, 1984; Menkes & Miclea, 1962; Menkes & Sandor, 1969; Packard Jr & Meier, 1984), the ‘clock and wavefront’ (Cooke & Zeeman, 1976) and the ‘cell cycle’ (Collier et al., 2000; Keynes & Stern, 1988; Primmett et al., 1988, 1989; Roy et al., 1999) models, as well as ‘reaction diffusion’, ‘clock and trail’ (Kerszberg & Wolpert, 2000; Meinhardt, 1982), the ‘wave and cell polarization’ model (Beloussov & Naumidi, 1983; Polezhaev, 1992) and the ‘progressive oscillatory reaction‐diffusion’ (PORD) model (Cotterell et al., 2015). Some molecular evidence apparently consistent with the ‘clock and wavefront’ model (Dubrulle et al., 2001; Dubrulle & Pourquie, 2004; Morelli et al., 2009; Oates et al., 2012; Palmeirim et al., 1997) has resulted in this model becoming dominant in the literature. However, it has also been argued that the ‘clock‐and‐wavefront’ model has been interpreted in various ways in the literature, resulting in several ‘sub‐models’ that are not identical to each other (Glazier et al., 2008; Hester et al., 2011). Among the proposals is the idea that a combination of cell‐repulsion and cell‐adhesion in adjacent cells can be coupled to the events of somite formation to explain the progression of boundary formation (Glazier et al., 2008, Hester et al., 2011).

Surprisingly, very few experimental studies have focused on cellular events within the PSM, and these have mainly examined aspects of the mesenchymal‐to‐epithelial transition (MET), performed cell tracking or studied extracellular matrix (ECM) assembly (Bellairs, 1979; Duband et al., 1987; Kulesa & Fraser, 2002; Martins et al., 2007, 2009). Some of the results of these studies are apparently contradictory, such as whether MET is initiated in the antero‐medial or the antero‐dorsal PSM (Beloussov & Naumidi, 1983; Kulesa & Fraser, 2002; Martins et al., 2009; Polezhaev, 1992). Most models of somitogenesis that have been proposed (see above) largely ignore the cellular dynamics within the PSM and generally assume that somite formation involves a sudden or ‘catastrophic’ MET causing cells to aggregate as a sphere. The main exceptions are the ‘wave and cell polarization’ model (Beloussov & Naumidi, 1983; Polezhaev, 1992) and three multi‐scale models (Dias et al., 2014; Glazier et al., 2008; Hester et al., 2011), all of which are based on various cellular properties. Here, we address the dynamics of cell shape changes, the progression of how cells aggregate into future somites, and the dynamics of cell polarization, using scanning electron microscopy (SEM), immunostaining and confocal microscopy. We find that the events of MET begin very posteriorly, therefore a long time before the formation of the respective somite. This implies that, rather than a sudden and catastrophic event just prior to the formation of a somite, epithelialization and cell polarization are gradual events. Moreover, these events are not exactly contemporary with each other because each appears at a distinct axial level within the PSM. A particularly surprising finding was that the different surfaces of the PSM undergo MET with different dynamics, rather than corresponding to the timing of formation of each somite. Since expression patterns of the ‘segmentation clock’ genes do seem to have boundaries covering the cross section of the PSM, these observations raise the question of how these gene expression patterns relate to the cellular events of somitogenesis.

## 2 MATERIALS AND METHODS

### 2.1 Embryos and scanning electron microscopy (SEM)

Fertilized domestic hens' eggs (*Gallus gallus*, Brown Bovan Gold, Henry Stewart & Co.) were incubated at 38°C to stages HH 11 (11–15 somites) (Hamburger & Hamilton, 1992). A newly formed somite 1 (s1) is defined as such when it is fully separated from the PSM, whereas somite 0 (s0) (at the tip of the PSM) is not separated by a completely formed cleft (Christ & Ordahl, 1995). In this study, we did not consider the first five somites (future occipital somites) because they form differently and have different structure to those of the trunk (Dias et al., 2014; Hamilton & Hinsch, 1956; Lim et al., 1987).

Embryos were harvested in PBS and processed for SEM as described (Bellairs, 1979), with the modification that after fixation in sodium cacodylate each embryo was cut only once, either transversely or sagittally, with a tissue chopper (Mickle Laboratory Engineering). PSM lengths were measured (see Supplementary Methods) and excess tissue was removed. Next, embryos were dried in CO_2_ (Leica CPD critical point dryer), mounted and sputter‐coated with gold/palladium. Images were taken on a JEOL JSM‐740IF Field Emission Scanning Electron Microscope (SEM) with 2000x magnification at 2KV and pressure of 5.25 10^−4^ Pa.

### 2.2 SEM image processing and aspect ratio analysis

Montages of the images were made using Photoshop CS6 and analysed with (FIJI) (Schindelin et al., 2012). A touch screen (SmartPodium 624) and touch pen were used to draw the outlines of each cell manually using the ‘freehand selection tool’ in FIJI. Only cells that were in focus and not covered by neighbouring cells were considered. Next, each cell outline was added to the Region of Interest (ROI) manager tool in FIJI. The aspect ratio (AR) for each cell was automatically calculated in FIJI by dividing the longest by the shortest dimension of the outline of a given cell as seen in the (two‐dimensional) SEM images. The AR values were colour‐encoded using a pseudo‐colour lookup table in FIJI. Assignment of cells to PSM or somite domains was done as described in Supplementary Methods (Section 2). The sagittal PSM fractures were mathematically straightened and variations in PSM length adjusted as described in Supplementary Methods (Section 3.1). Then, for each domain separately, the AR of cells, represented as points, were plotted against PSM distance represented as a percentage of the total length of the PSM in the same embryo. The most posterior PSM is called 0% and the PSM‐somite border is designated as 100%. Next, a curve was fitted using regression analysis (see Supplementary Methods, Section 3.3). For transverse fractures, the AR of cells was represented in box plots for each domain at specific PSM positions (see Supplementary Methods, Section 3.2).

### 2.3 Whole‐mount immunostaining

Embryos were harvested in PBS, pinned to a silicon‐coated Petri dish and excess tissue trimmed with a blade. For GM130 (1:100, mouse, BD Biosciences #610822) and N‐cadherin (1:100, mouse, BD Biosciences, #610921) antibody staining, fixation was in 4% Paraformaldehyde (PFA) at room temperature for 15 min. For PKCζ (1:100, rabbit, SantaCruz, SC216), PAR3 (1:300, rabbit, Upstate, 07330) and ZO1 (1:50, rat, SantaCruz, SC33725) antibody staining, embryos were fixed in 1% PFA for 15 min at 4°C. Three embryos per marker were used except for ZO1 where two embryos were analysed. After fixation, embryos were permeabilized overnight in PBST (1% Triton‐X100 in PBS) with 0.02% thimerosal and blocked for a day at 4°C with 0.1% BSA (Sigma, A3803‐50G) with 5% heat‐inactivated goat serum (Sigma, G6767) in PBST. Then, embryos were incubated in blocking solution with the desired primary antibody and washed several times overnight in PBST. They were then incubated in the dark for 1 day in blocking solution with the appropriate secondary antibody: Alexa 488‐conjugated anti‐mouse (Life Technologies, A21202), anti‐rabbit (Life Technologies, A11008) or anti‐rat (Life Technologies, A11006) together with 1:2000 ToPro3 nuclear stain (Molecular probes, T3605) and 10 μg/mL RNase A (Sigma, R6513). Finally, embryos were cleared and mounted for imaging (see Supplementary Methods).

### 2.4 3D imaging, processing and analysis

3D Images were acquired with an Olympus Fluoview FV1000 confocal microscope and processed in FIJI (see Supplementary Methods). For each sample, the image was optically re‐sliced in the transverse plane and the PSM was divided into medial, lateral, dorsal and ventral domains, with each domain further divided into apical and basal halves (for details see Supplementary Methods). Next, the pixel intensity differences between apical and basal compartments were calculated for each domain and plotted against PSM distance represented as 0%–100% (see above and Supplementary Methods). To identify when the observed changes occur, two embryos with DiI‐labelled cells were followed by time‐lapse filming and the positions of labelled cells were recorded, allowing scaling of PSM position in relation to real developmental time (see Supplementary Methods).

### 2.5 Whole‐mount in situ hybridization

Embryos were fixed in 4% PFA and stored in methanol at −20°C. They were then bleached for 1 h in 6% H_2_O_2_ in methanol. In situ hybridization was carried out as described (Streit & Stern, 2001) using digoxigenin‐labelled probes: Paraxis (Burgess et al., 1995; Burgess et al., 1996), LFng (Sakamoto et al., 1997), Hairy 1 (Palmeirim et al., 1997), Meso2 (Buchberger et al., 2002), EphA4 (Gilardi‐Hebenstreit et al., 1992). The Uncx4.1 probe (Dale et al., 2003; Neidhardt et al., 1997) was prepared by PCR with GOTaq (Promega) to amplify the uncut plasmid, with primer pairs M13F: GTAAAACGACGGCCAGT, M13R: GCGGATAACAATTTCACACAGG for 30 cycles with 1 min at 50°C for annealing.

### 2.6 Sectioning and imaging

Images of whole‐fixed embryos were acquired with an Olympus SZH10 Stereo microscope equipped with a Q‐Imaging Retiga 2000 K camera, controlled using QCapturePro software. The embryos were positioned as desired in depression slides in PBS. Next, embryos were embedded in paraffin wax and sectioned sagittally at 10 μm using a microtome (Microm HM 315). They were mounted in 3:1 Canada Balsam (Merck KGaA, 8007‐47‐4): histoclear (HS‐202 HISTO‐CLEAR II, National Diagnostics) and imaged with an Olympus VANOX‐T AH2 microscope with 40x oil immersion objective (NA 0.7).

### 2.7 Hybridization chain reaction

Embryos were fixed in 4% PFA and stored overnight at −20°C in methanol. Hybridization Chain Reaction (HCR) (Dirks & Pierce, 2004) was performed according to the HCR v3.0 protocol from the manufacturers (Molecular Instruments) for whole‐mounted chicken embryos. Embryos were mounted on glass slides using SecureSeal 13 mm × 0.12 mm imaging spacers (Grace Bio‐Labs). A drop of SlowFade Gold (Invitrogen) was placed on the embryo, which was then covered with a No. 1 coverslip. Embryos were imaged using a Leica SP8 laser scanning confocal microscope with an Apochromat 40X 1.4 NA oil objective. 3D maximum projections and sagittal sections were generated using Imaris 8.2 (Bitplane).

## 3 RESULTS

### 3.1 Cell shape changes during somitogenesis

#### 3.1.1 Cell shape changes in sagittally fractured PSM

**FIGURE 1:**
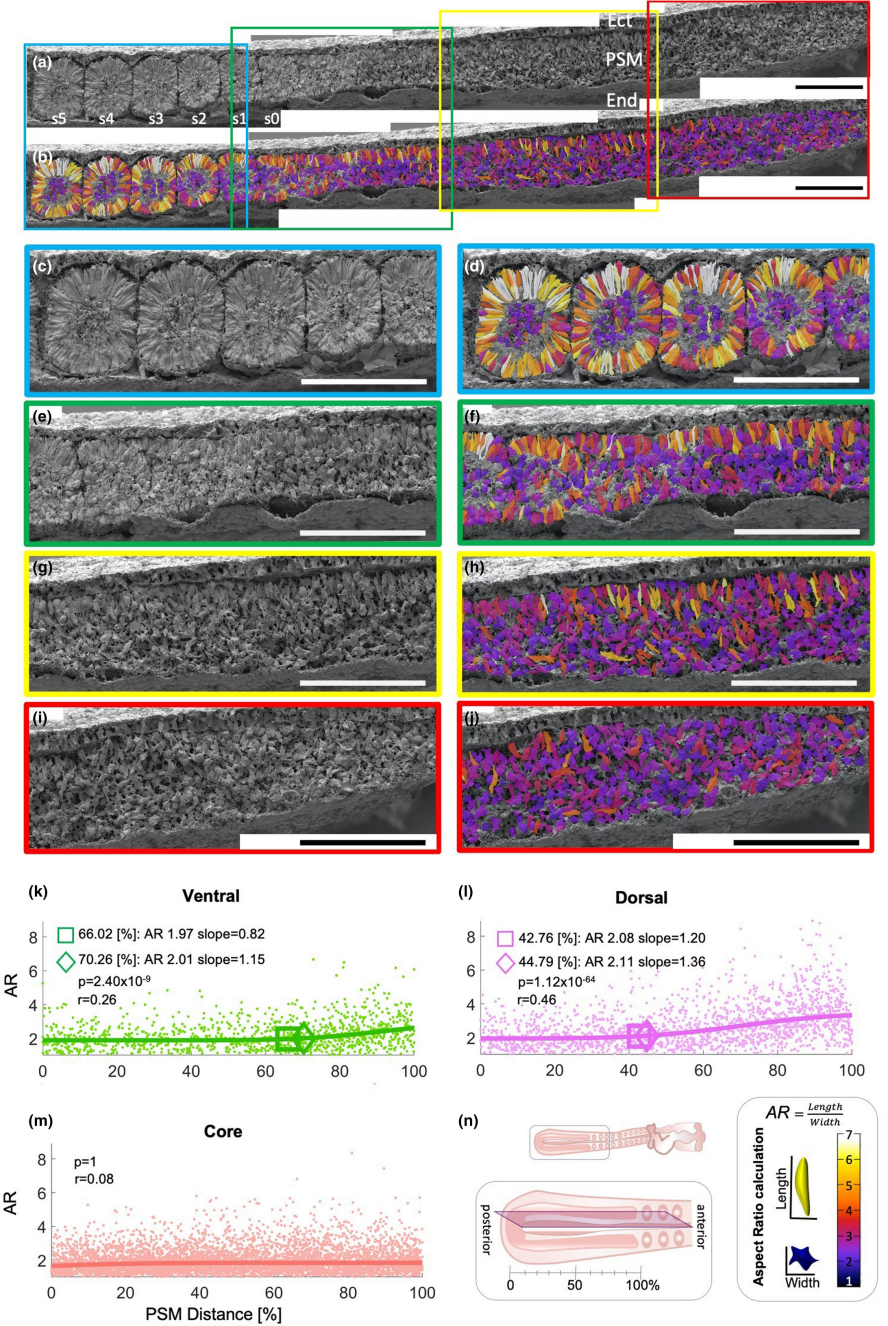
Aspect ratio changes along the midsagittal PSM. (a) An example of midsagittal PSM and somite fracture, s1 is the 12th somite. (b) The same sample with the cell outlines colour coded for their AR. (c–j) Enlargements of the same sample as indicated in coloured boxes in (a, b). Scale bars are 100 μm. S ‐ somite, PSM‐ pre‐somitic mesoderm, Ect – ectoderm, End ‐ endoderm. (k–m) Aspect ratio vs distance in sagittal PSM sections. The ARs of each cell from 12 embryos were plotted against distance along the PSM represented in [%]. Each dot represents the AR of a single cell allocated to a domain. Lines represent a sigmoid curve fitted with regression analysis. The diamond is an inflection point; the square is the point of 10% of vertical height of the fitted curve. *p*‐values are calculated with *F*‐test. (n) Schematic representation of a midsagittal fracture with posterior‐PSM represented as 0% of its length and anterior‐PSM as 100%. Aspect ratio calculations and colour coding shown on the bottom right pan.

A total of 8318 cells from 12 mid‐sagittally fractured embryos were classified as belonging to the dorsal or ventral PSM domains or to the core (Supplementary Table S1) and AR frequency distributions per domain were plotted as histograms (see Supplementary Figure S1). An example of a mid‐sagittally fractured embryo, with cells colour‐coded for their AR, is presented in Figure 1a–j. Dorsal‐PSM cells elongate in the posterior‐PSM (AR ≥5), which is earlier than those of the ventral‐PSM (Figure 1j). There are occasional clusters of epithelialized cells in the dorsal‐PSM (Figure 1h: AR = 6). The cells of the ventral‐PSM have AR ≥5 in the anterior‐PSM, prior to somite formation (Figure 1f). The core‐PSM cells remain mesenchymal (Figure 1c–j). Some core cells in the posterior‐PSM have aspect ratios of 4–5, but they have long protrusions (Figure 1h,j).

To study MET changes for each domain along the mid‐sagittal PSM, AR values were plotted against PSM distance [0%–100%] (Figure 1k–m). Two indicators were used to determine at what PSM distance [%] a change in epithelialization rate occurs: the inflection point (Figure 1k–m, diamonds) and 10% of vertical height of the fitted curve point (Figure 1k–m, squares). The AR of cells in the dorsal domain increases at 44% of the PSM distance, as indicated by the inflection point, or at 42% as indicated by the 10% of vertical height point (*F*‐test *p* = 2.40 × 10^−9^), whereas the AR for the ventral domain increases after the dorsal domain at 70% and 66% for each point respectively (*p* = 1.12 × 10^−64^). The AR of core cells does not change with distance along the PSM (*p* = 1). Thus, for the sagittally fractured PSM, the order of epithelialization as indicated by AR is as follows: first dorsal at 40% of PSM distance, then ventral at about 70%; core cells never epithelialize.

#### 3.1.2 Cell shape changes in transversely fractured PSM

**FIGURE 2:**
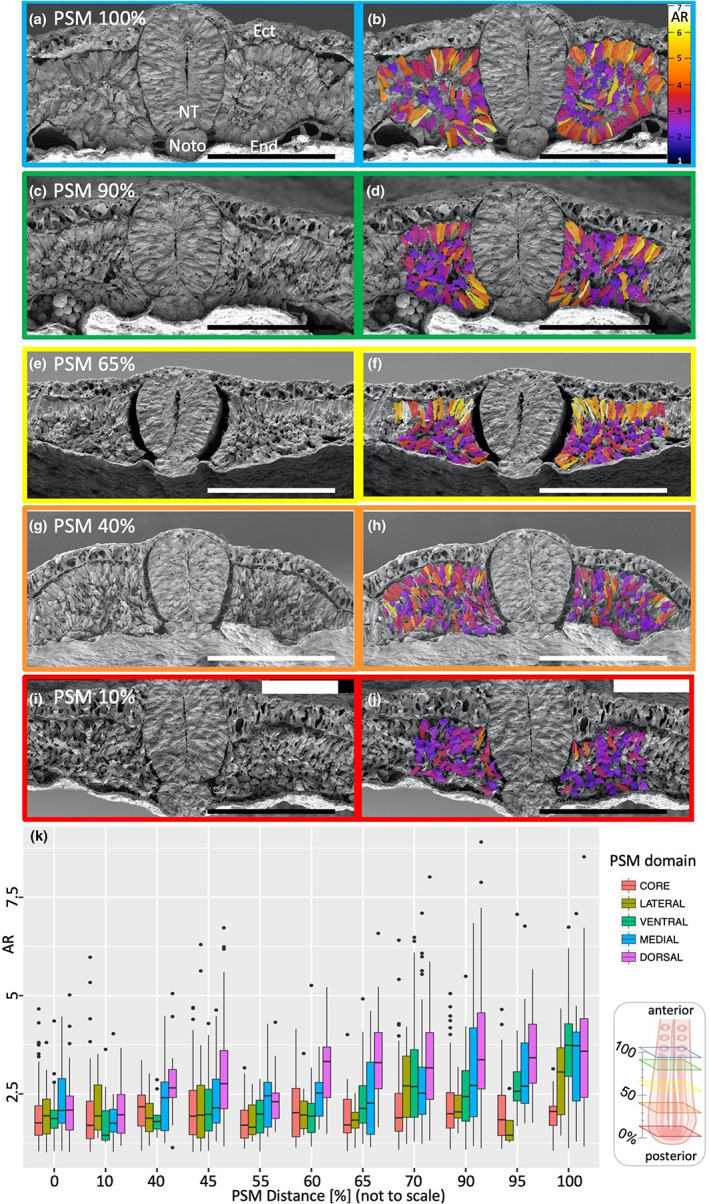
SEM images of transversely fractured PSM at different distances. (a, c, e, g, i) transverse fracture at 100%, 90%, 65%, 40%, and 10% of PSM, (b, d, f, h, j) corresponding AR colour coding of the same embryos. Each image is a different embryo. Scale bars are 100 μm. Ect – ectoderm, end – endoderm, NT‐ neural tube, Noto ‐ notochord. (k) a box plot of aspect ratio per domain versus distance in transverse PSM fractures. Insert indicates schematic representation of PSM transverse fractures at different distances along the PSM distance [%]. Boxes are colour coded and correspond to (a–j).

For the transverse PSM fractured embryos, 2267 cells from 20 embryos were assigned to dorsal, ventral, medial, lateral or core domains (Supplementary Table S2). Examples of transversely fractured embryos at different rostrocaudal levels of the PSM are shown in Figure 2. In the most anterior PSM (100% distance; Figure 2a,b), the elongated cells at AR = 4–7 form a rosette and display epithelial morphology in all PSM domains except the core, where cells are mesenchymal (AR = 1–4). The observation that the rosette is not fully closed in the lateral PSM suggests that a newly forming somite separates not only from the PSM but also from more lateral mesoderm. Slightly more caudally, at 90% of the PSM (Figure 2c,d), some elongated cells of high AR are present in all domains but are very scarce in the lateral PSM and in the core. At the 65% position (Figure 2e,f), high AR cells are observed mainly dorsally with occasional elongated cell clusters appearing in the medial and ventral surfaces of the PSM. At 40% of the PSM (Figure 2g,h) the predominant cell morphology is mesenchymal (low‐AR) in all domains; higher AR cells are rare and mostly situated dorsally. At 10% of PSM distance, elongated cells of AR 4–5 are present only occasionally in dorsal and medial domains and the remaining cells are mostly mesenchymal (Figure 2i,j).

Cells of non‐epithelial morphology with long protrusions are found in the core at all PSM levels, suggesting that these cells may be motile, consistent with previous reports of cell movement both within and between somites (Kulesa et al., 2007; Kulesa & Fraser, 2002; Martins et al., 2009), and in the PSM (Benazeraf et al., 2017).

AR values for cells in each domain at different positions in the PSM [%] are represented in a box plot (Figure 2k). The lateral domain has a relatively low number of cells (see Supplementary Table S2) because it was defined as a single layer of cells that is neither dorsal nor ventral nor core, and therefore comprises relatively few cells. Despite this, clear trends are apparent from the plots. The AR of cells in the dorsal domain starts to increase very early, at 40% of the PSM length, consistent with the results from the sagittally fractured PSM. For medial cells, the AR starts to increase at 55% PSM. In both the ventral and lateral domains, AR starts to increase at 70%, also consistent with the sagittal sections. Cells in all four domains (excluding the core) increase their AR with PSM position (*t*‐test *p* = 2 × 10^−16^). The cells of the core domain do not increase in AR at any PSM position (*t*‐test *p* = 0.016). No significant differences were found between the left and the right PSM at each position (*t*‐test *p* = 0.608). Statistical analyses (*t*‐test) of these comparisons are given in Supplementary Table S3 (red denotes a significant difference).

In summary, cells located at the edges of the PSM (dorsal, lateral, ventral, and medial) begin to undergo elongation (increase in AR) at different rostro‐caudal levels of the PSM: dorsal cells start elongating first (40%), followed by medial cells (55%), and finally the ventral and lateral domains, which undergo this transition at the same time (70%). In contrast, the core cells appear to be trapped between epithelializing cells, and never elongate except for occasional cells with long protrusions.

#### 3.1.3 Cell shape changes in sagittally fractured somites

**FIGURE 3:**
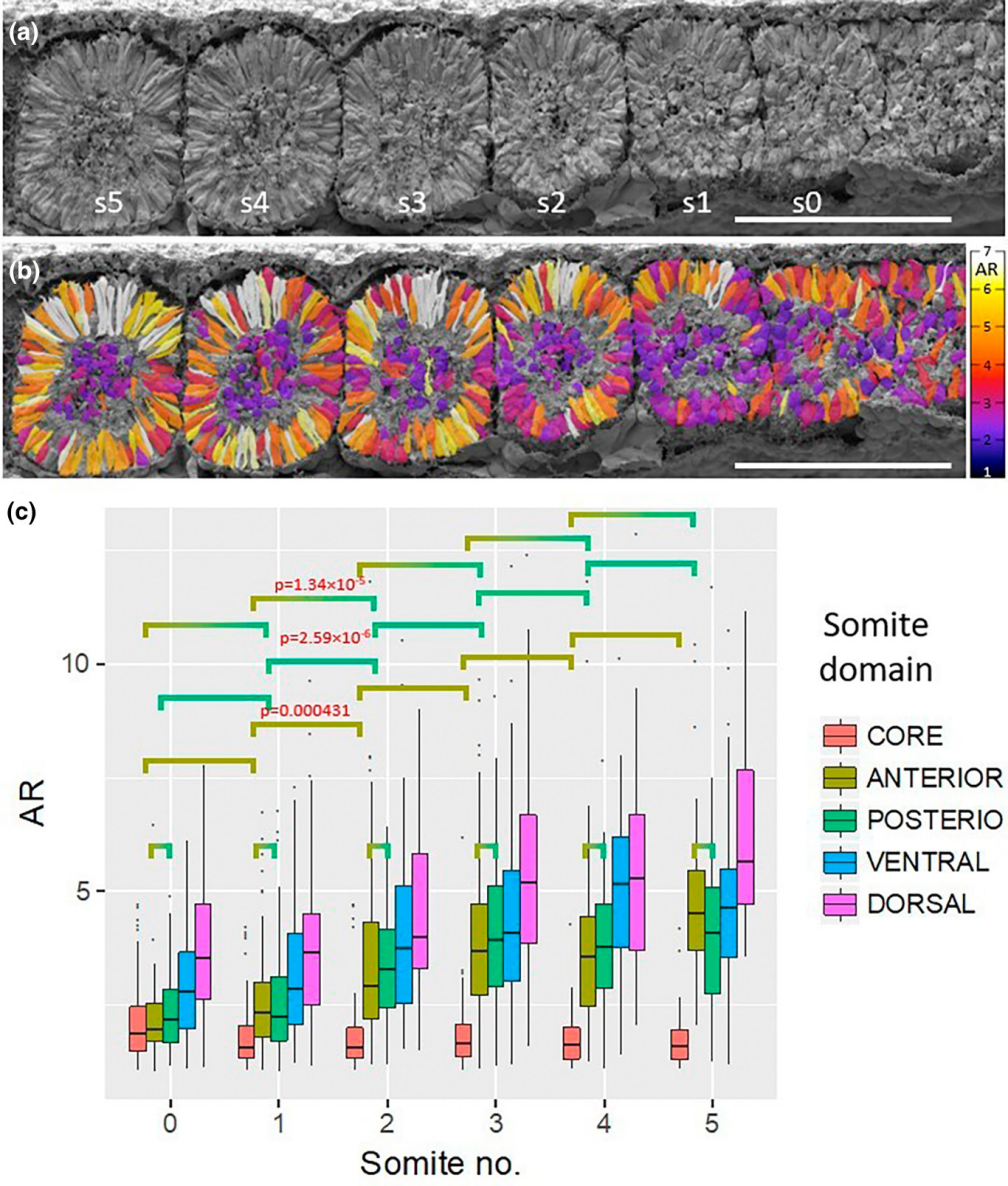
SEM images of mid‐sagittal fractures of somites. (a) an embryo with newly formed somites (s0‐s5). (b) the same embryo shown with the cell outlines colour coded for their aspect ratio (insert in b is colour coded for AR scale). Scale bars represent 100 μm. (c) Box plot of aspect ratio versus somite number in sagittal somite fractures. *p*‐values from two‐sided *t*‐test for domain comparisons within a somite are listed in Supplementary Table S5 (red is significant). Comparisons of the same domain for consecutive somites are listed in Supplementary Table S6.

Among the 12 embryos that were fractured sagittally through the segmental mesoderm, several fracture planes included the newly formed somites (s0‐s5). In total, 2180 somite cells were analysed and assigned to dorsal, ventral, anterior and posterior domains and the somitic core (somitocoele) (Supplementary Table S4). Histograms of AR frequency distributions per domain for each somite number were plotted (Supplementary Figure S2). A representative mid‐sagittal fracture of somites s0‐s5 is shown in Figure 3a,b. These somites have core cells with mesenchymal morphology (AR 1–4), but there are occasional core cells with long protrusions (e.g. s3, s4). Somites s3‐s5 are epithelial rosettes with most cells having epithelial morphology and an AR between 5 and 7 in all domains except the core. The anterior and posterior aspects of somite s2 have an equally low AR (2–3), which is lower than in the dorsal and ventral domains. However, in s1 the cells of the anterior domain have a lower AR of 2–3 compared to the cells in the s2 posterior domain, which have an AR of 4–7. In s0 most of the high‐AR cells are located dorsally, with some in the ventral and posterior domains.

The AR values of each domain for each somite position are plotted in Figure 3c. Comparison between domains within a given somite was done with a Wilcoxon test (Supplementary Table S5: red denotes a significant difference). No significant difference in AR was found between the anterior and posterior domains for all somite positions. There is a significant difference between the AR of core cells and all other domains for all somites, except in the anterior and posterior domains of s0 (the somite in the process of separating from the PSM). The borders of s0 are not fully formed and the AR of anterior and posterior cells resembles the AR of the core cells. However, there is a significant difference between the dorsal domain and the anterior and posterior domains for all somites. The ventral domain is significantly different from the anterior domain for s0 and s1. There is a significant difference between the ventral and dorsal domains for s0, probably because the dorsal domain epithelializes more posteriorly than the ventral domain within the PSM, hence the dorsal somitic cells already have a higher AR, and the ventral cells are ‘catching up’. However, cells in the ventral and posterior domains of s1, but not in the dorsal domain of the same somite, display a significant AR difference. In s4 and s5, there is a significant difference between the ventral and posterior domains, and the ventral and dorsal domains respectively. This difference could be due to the presence of occasional short spindle‐shaped cells in those locations (Figure 3b). Comparison (*t*‐test) between a given domain across different somite positions revealed a significant increase in AR for all domains (except the core) with increasing somite number (Supplementary Table S6). This result suggests that the cells of the epithelial walls of the somite continue to elongate, while the core cells remain mesenchymal. Wilcoxon pairwise comparison of consecutive somites revealed significant differences between the anterior domains of s1 and s2, and also between the posterior domains of these somites (Supplementary Table S7). This is because somites 0 and 1 do not have cells of high AR in their anterior and posterior domains, but the next somite, s2, has more elongated cells in its anterior and posterior domains, and more rostral (older) somites have highly elongated cells in those domains (Figure 3a,b). As no significant difference was found between the anterior and posterior domains within each somite, the anterior and posterior domains of consecutive somites were compared with a two‐sided *t*‐test (Figure 3d). A significant difference was found between the anterior domain of s1 and the posterior domain of s2 (*p* = 1.34 × 10^−5^).

Collectively, the results from sagittally and transversely fractured PSM suggest that MET, as indicated by increase in AR, starts at the caudal end of the PSM at about 40% of the length of the PSM. Therefore, these cellular changes occur gradually long before a somite buds off from the PSM, starting from the most dorsal cells, followed by those placed medially, and finally those in the ventral and lateral aspects of the PSM, like a long rectangular box gradually being constructed by laying down the roof, then one side, then the floor, and finally the other side before cutting it into cubes. The anterior and posterior cells of a somite elongate only after the border is formed. The border between a newly formed somite and the anterior PSM resembles a ball and socket, as previously described (Kulesa & Fraser, 2002; Martins et al., 2007). The posterior domain of the preceding somite contains more elongated cells than the anterior domain of the subsequent somite, which may indicate how the border is maintained.

### 3.2 Dynamics of cell polarity changes during somitogenesis

To obtain a 3D view of how cell polarity changes within the PSM as cells progress towards forming a somite, whole‐mount embryos were stained for cell polarity markers GM130 (a Golgi marker), PAR3, ZO1 and PKCζ, and N‐cadherin (the latter as an ECM marker). For each marker, coronal, sagittal and transverse plane views of the 3D images are presented in Supplementary Figures S3–S12.

**FIGURE 4:**
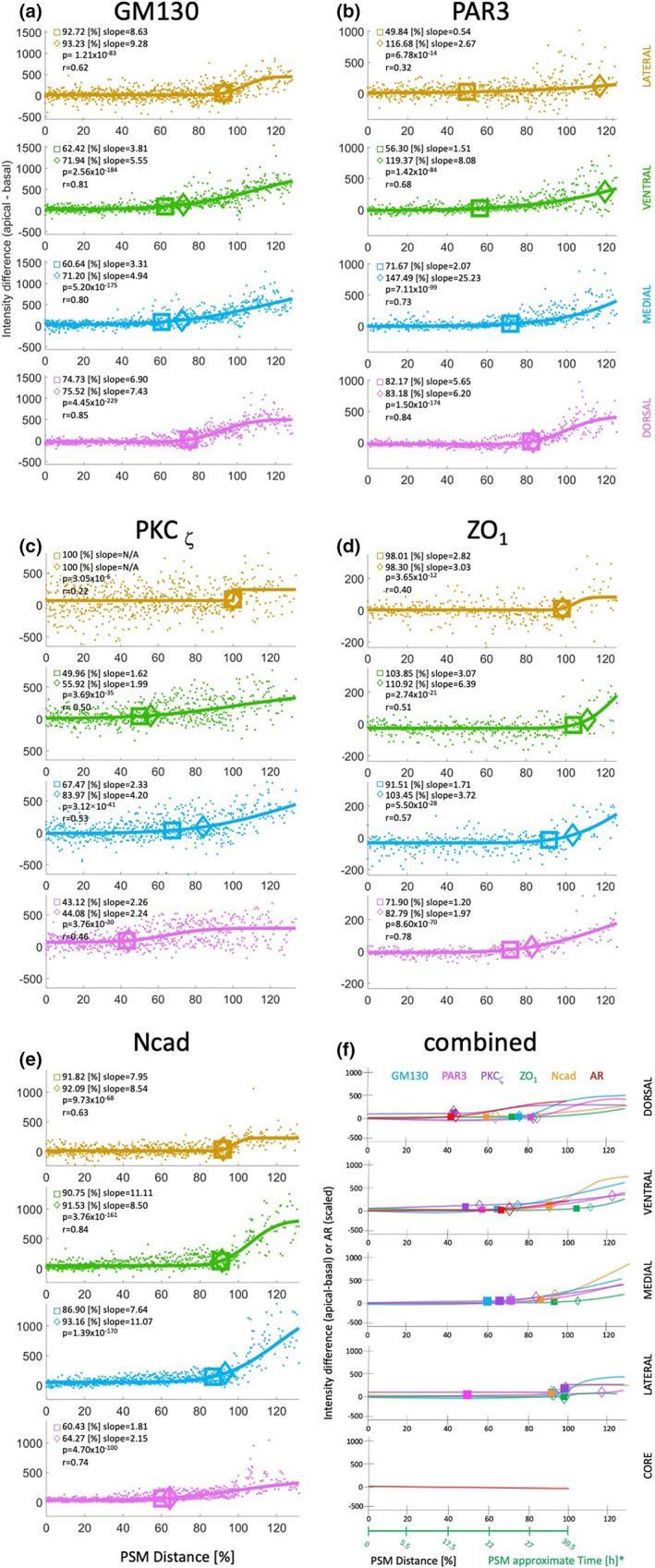
Pixel intensity differences of polarity markers versus PSM distance [%]. (a) GM130 (b) PAR3 (c) PKCζ (d) ZO1 (e) Ncad (f) combined epithelialization and polarity data with time rescaling. For each marker, pixel intensities of the basal zone were subtracted from those of the apical zone for every PSM domain and plotted against PSM distance represented in [%]. 0%–100% is PSM, whereas values above 100% represent somites. Each dot is a single data point (apical‐basal). The line is a sigmoid fit from regression analysis. Squares represent 10% of vertical height of the fitted curve and diamonds are inflection points.

The Golgi apparatus is positioned in front of the nucleus, therefore the alignment between these two organelles defines a cell directionality vector. The cell density in the anterior PSM and somites is too high to assign the Golgi apparatus to specific nuclei with confidence. Therefore, analysis of Golgi position at the cell population level was used to reveal an overall pattern. If the overall population is not polarized then the Golgi should be scattered in both apical and basal zones of each domain, whereas polarized cells should have the Golgi apparatus aligned in one zone. Staining for GM130 (green) along with TOPRO nuclear staining (purple) revealed that the Golgi ribbons are positioned near the centre of the somite (Supplementary Figure S3). The forming somite forms a ‘basket’ with its anterior domain not fully closed by the ring of Golgi structures (Supplementary Figures S3B,E, Figure 4b,c,e,f). The Golgi apparatus is aligned with the nuclei in the dorsal, medial and ventral domains of the anterior‐PSM (Supplementary Figure S4B,C,F). Transverse views most clearly show the full ring of Golgi structures around the somites (Supplementary Figure S4A–C) and very anterior PSM (Supplementary Figure S4D–F). The ring gradually ‘opens’ in the lateral PSM (Supplementary Figure S4–S12G–I); more posterior parts of the lateral domain have scattered GM130 labelling. Still further posteriorly (Supplementary Figure S4J–L), the Golgi ribbon and nuclei start to lose their linear alignment in the dorsal, medial and ventral domains, until, in the most posterior PSM (Supplementary Figure S4M–X), the cells of all domains appear to be randomly oriented.

Like GM130, staining with PAR3 reveals a ring structure in the centre of formed somites (Supplementary Figures S5 and S6). Coronal (Supplementary Figure S5A–F) and sagittal views (Supplementary Figure S5G–I) show that the forming somites have very little PAR3 signal in the anterior domain. There is weak expression of PAR3 in the PSM (Supplementary Figure S5A–I). Transverse views show a full ring of PAR3 in the apical zone of the dorsal, ventral, medial and lateral domains of the newly formed somite (Supplementary Figure S6A–C). The intensity of staining and the ring structure gradually decrease posteriorly until at the most posterior levels of the PSM, there is almost no expression of PAR3 in any domain and no obvious pattern (Supplementary Figure S6J–X).

PKCζ staining also reveals a ring pattern in the centre of the somites that have already separated from the PSM and in the newly forming somites (Supplementary Figures S7A–F and S8A–F). However, unlike the patterns of the previous two markers, all cells of the PSM and somites express PKCζ in the cytoplasm before PKCζ becomes polarized. This ring‐arrangement starts to disappear just posterior to the newly forming somites (Supplementary Figure S8G–I), with no obvious polarization in more posterior regions of the PSM (Supplementary Figure S8J–X).

The zona occludens marker ZO1 resembles PKCζ more than the previous markers: staining reveals a ring structure in the apical parts of cells in all domains of the forming and newly formed somites (Supplementary Figures S9 and S10). In the forming somite, the apical staining is weaker in posterior and dorsal domains. Further posteriorly, in the PSM, there is no obvious organized pattern of ZO1; staining is distinct and cytoplasmic throughout the PSM, apart from its anterior end, as it becomes localized apically to generate the distinct ring in the forming somite.

N‐cadherin (Ncad) is a membrane‐associated protein that is deposited on the apical side of epithelial cells. Its localization is most pronounced at the level of the formed somites, where it is predominantly present in the somitocoele (Supplementary Figures S11 and S12). At the level of the forming somite, there is Ncad at the apical zone of the dorsal, ventral and posterior domains but not in the anterior domain (Supplementary Figure S11D–F), a pattern referred to as ‘the basket’ in a previous report (Martins et al., 2009). In the anterior PSM, Ncad is localized dorsally and ventrally (Supplementary Figure S11D–F). Maximum intensity projections show expression through the entire PSM with a slightly higher intensity localized medially (Supplementary Figure S11A–C), followed more posteriorly by a thin line of medial staining (Supplementary Figure S11A–C). There is no organized staining pattern at more caudal levels of the PSM (Supplementary Figure S11A–F).

Transverse optical sections were used to quantify the observed changes in distribution patterns of each marker. The intensity plots of the difference between apical and basal compartments of each domain were plotted against PSM position [%] for each marker (Figure 4). Coordinates of the inflection (squares) and 10% of vertical height (diamonds) points are shown next to these points respectively. F‐tests were used to calculate p‐values at characteristic positions in each domain (see Supplementary Methods). The slopes of the curve for each domain and marker indicate how fast polarization happens. Correlation coefficients reflect the relationship between polarization and PSM position. The core was not analysed because apical and basal domains cannot be defined for its mesenchymal cells.

PAR3 expression in the lateral and ventral domains polarizes first at 50% and 60% of PSM distance, respectively, as indicated by 10% of vertical height (Figure 4a, squares). The medial domain epithelializes at 70% as indicated by both the inflection point (diamond) and the 10% of vertical height (square). The shallow slope of the curves suggests that polarization of PAR3 occurs very gradually. The dorsal domain starts to polarize more steeply at about 80% of PSM position.

For PKCζ (Figure 4b), the dorsal domain starts to polarize first at 40% of PSM position, followed by the ventral domain at 50%, medial at 65% (square) and 85% (diamond) and finally the lateral domain at 100% (somite formation). These observations suggest that the lateral domain epithelializes very rapidly at the PSM‐somite boundary, whereas the other domains undergo gradual epithelialization.

The dorsal domain of ZO1 (Figure 4c) polarizes first at 70% (square)—85% (diamond) of PSM position. The medial domain follows at 90%–105%, the lateral at 100% (forming somite); the ventral domain only polarizes after the somite has fully budded from the PSM.

Quantification of GM130 polarization (Figure 4d) reveals that the ventral and medial domains polarize simultaneously and earliest, at about 60% (square) and about 70% (diamond) of the PSM length. The dorsal domain then starts to polarize more rapidly at about 80% of the PSM; the last domain to polarize is the lateral aspect, just prior to somite formation at about 95% of the PSM.

Ncad staining (Figure 4e) reveals that the dorsal domain starts to polarize at 60% of the PSM and gradually increases anteriorly. The medial, ventral and lateral domains epithelialize almost simultaneously at about 90% of PSM; thereafter epithelialization occurs rapidly, as suggested by the steep slope, consistent with a previous report (Duband et al., 1987).

In summary, cells in the dorsal, medial, lateral and ventral domains of the segmental mesoderm polarize very gradually. For a given polarity marker, each domain starts the process at a different level of the PSM and undergoes polarization at a different rate (and with characteristic patterns for each marker). Our findings are summarized in Figure 4f, which combines our results on MET (as indicated by change of AR) and cell polarization (indicated by GM130, PKCζ, PAR3, ZO1 and Ncad staining). The earliest events of polarization (PKCζ) and epithelialization (AR) are observed in the dorsal domain at around 40% of the rostro‐caudal length of the PSM. Then polarization begins ventrally at 50% of the PSM, prior to epithelialization at about 65% of the PSM, followed by lateral polarization, and finally followed by the medial domain.

### 3.3 Relationship between PSM position and the timing of segmentation

**FIGURE 5:**
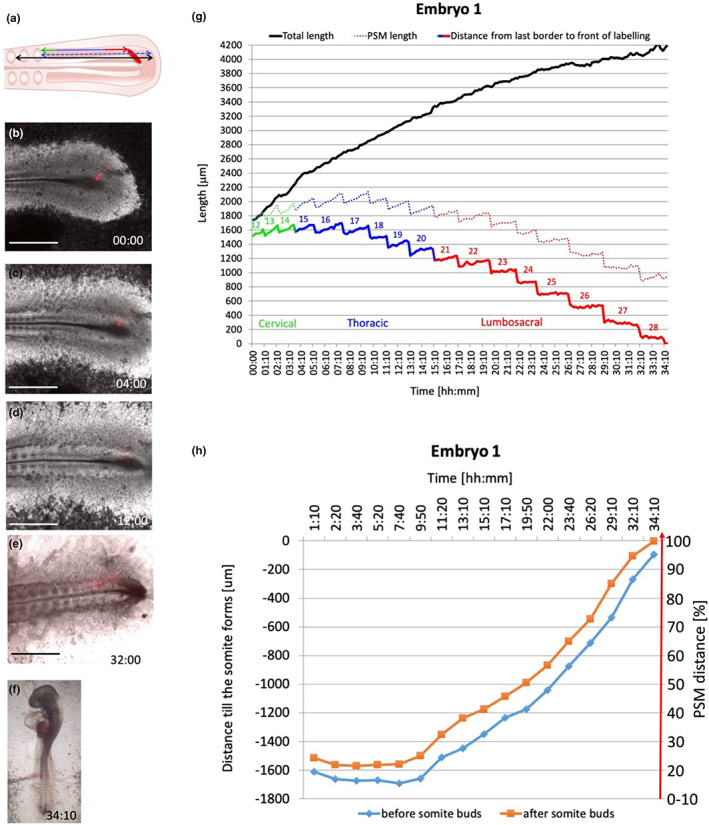
Time‐lapse imaging of somitogenesis ‐ embryo 1. (a) the diagram represents how the distances within the PSM were measured. (b–e) somite formation at different time points, (f) embryo morphology at the end of the experiment, (g) relationship between position along the PSM and the time elapsed since cells were located at the inferred posterior end of the PSM. (h) Plot of time elapsed since the labelled cells reached the somites. Scale bars are 1000 μm. See Supplementary Movie S1.

**FIGURE 6:**
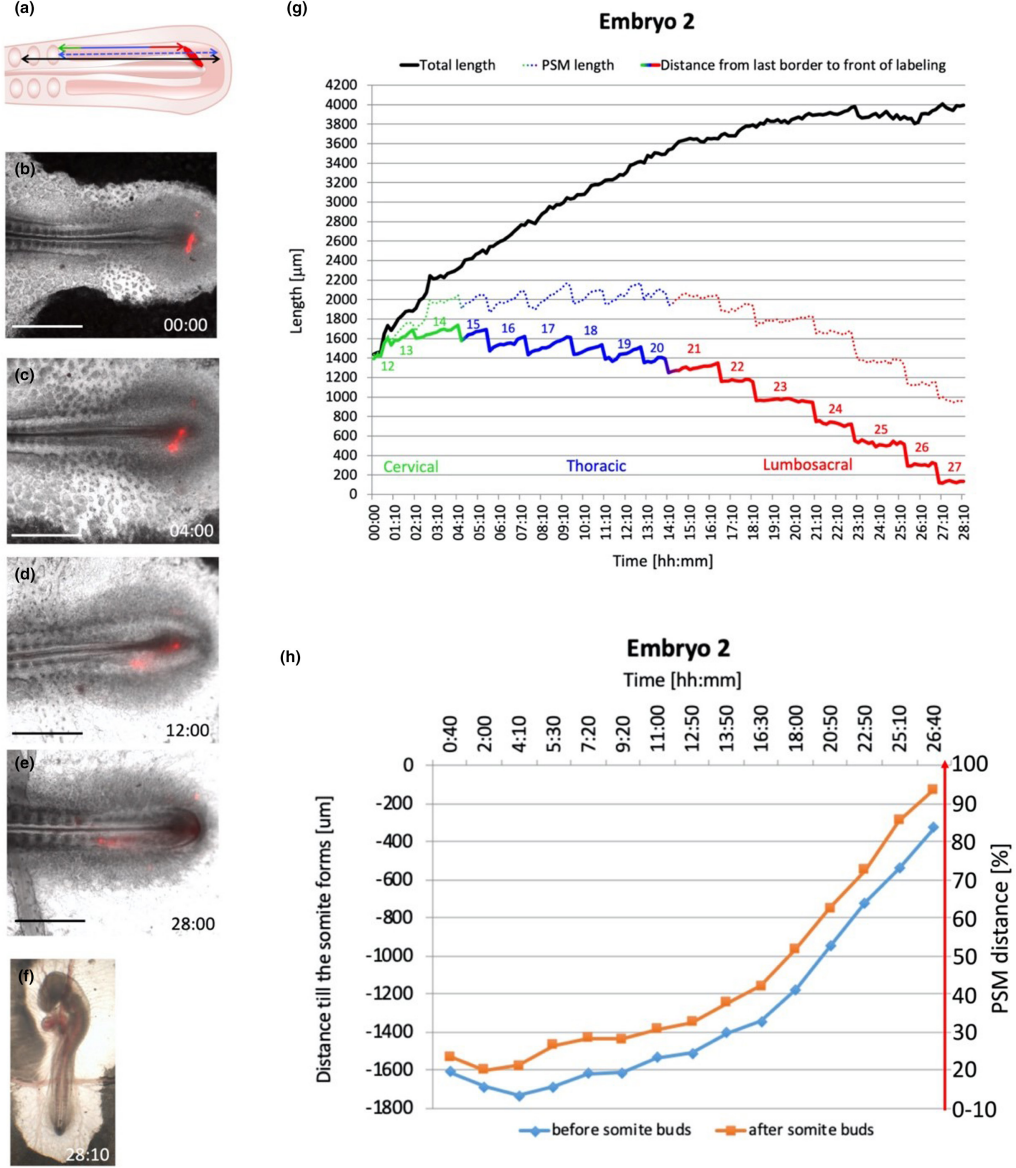
Time lapse imaging of somitogenesis embryo 2. (a) the diagram represents how the distances within the PSM were measured. (b–e) somite formation at different time points. (f) Embryo morphology at the end of the experiment. (g) Relationship between position along the PSM and the time elapsed since cells were located at the inferred posterior end of the PSM. (h) Plot of time elapsed since the labelled cells reached the somites. Scale bars are 1000 μm. See Supplementary Movie S2.

The relative rostro‐caudal position of a given cell within the PSM is also a function of time elapsed since the cells entered the PSM from the primitive streak, and consequently of the time remaining for those cells before segmentation occurs. Because the PSM is a spatiotemporally organized system, it is possible to translate the position of a given event occurring within the PSM into the approximate time at which that event happens. The relationship between position and time was determined with time‐lapse video microscopy of embryos in which cells just entering the caudal PSM had been labelled with the fluorescent dye, DiI. Two embryos that developed well for a sufficient period were analysed: embryo 1 (Figure 5 and Supplementary Movie S1) and embryo 2 (Figure 6 and Supplementary Movie S2). For each time point, three distances were measured (Figures 5 and 6a): (1) the total length of the segmented mesoderm (PSM and somites formed during the experiment) was plotted over time (Figures 5 and 6a,g black line), (2) the distance from the DiI front to the somite border (Figures 5 and 6a,g rainbow line) and (3) the length of the PSM from the somite border to the posterior‐PSM (Figures 5 and 6a,g rainbow dotted line). The rate of somite formation in these embryos averaged 68 min per somite (Embryo 1 formed 28 somites in 34 h 10 min and embryo 2 formed 27 somites in 28 h 10 min). Both embryos displayed a gradual increase in the total amount of somite tissue (Figures 5 and 6a,g black), slowing down as somitogenesis begins to take place, as previously described (Gomez et al., 2008). The distance from the most caudal (most recently formed) somite border to the front of the labelled cells was plotted against time in reverse order to follow how soon the labelled group of cells contributed to somites, and whether this rate was constant (Figures 5 and 6h). Despite the difference in the rate and overall number of somites formed for the two embryos, both embryos displayed the same trend: initially cells progressed slowly along the PSM before starting to accelerate, from about 10 h for embryo 1 and 11 h for embryo 2. After the initial 10–11 h, the rostral progression of cells in the PSM sped up at a constant rate (Figures 5 and 6h). This is consistent with previous studies (Benazeraf et al., 2017; Selleck & Stern, 1991; Stern et al., 1988) which suggest that cells intermingle extensively in the caudal PSM and then gradually stop doing so as they become located more rostrally. This new analysis therefore provides an approximate translation of PSM position (distance) at which a given event occurs into the time at which that event happens. The PSM distance [%] scale on the y axis represents the distance along the PSM to the level of the forming somite [μm] (Figures 5 and 6h), and the X coordinates from the inflection and 10% distance points were used to extrapolate PSM position to the timing of events within the PSM. Figures 5 and 6h summarizes these findings.

### 3.4 Do the rostral and caudal subdivisions of the somite extend to the core?

**FIGURE 7:**
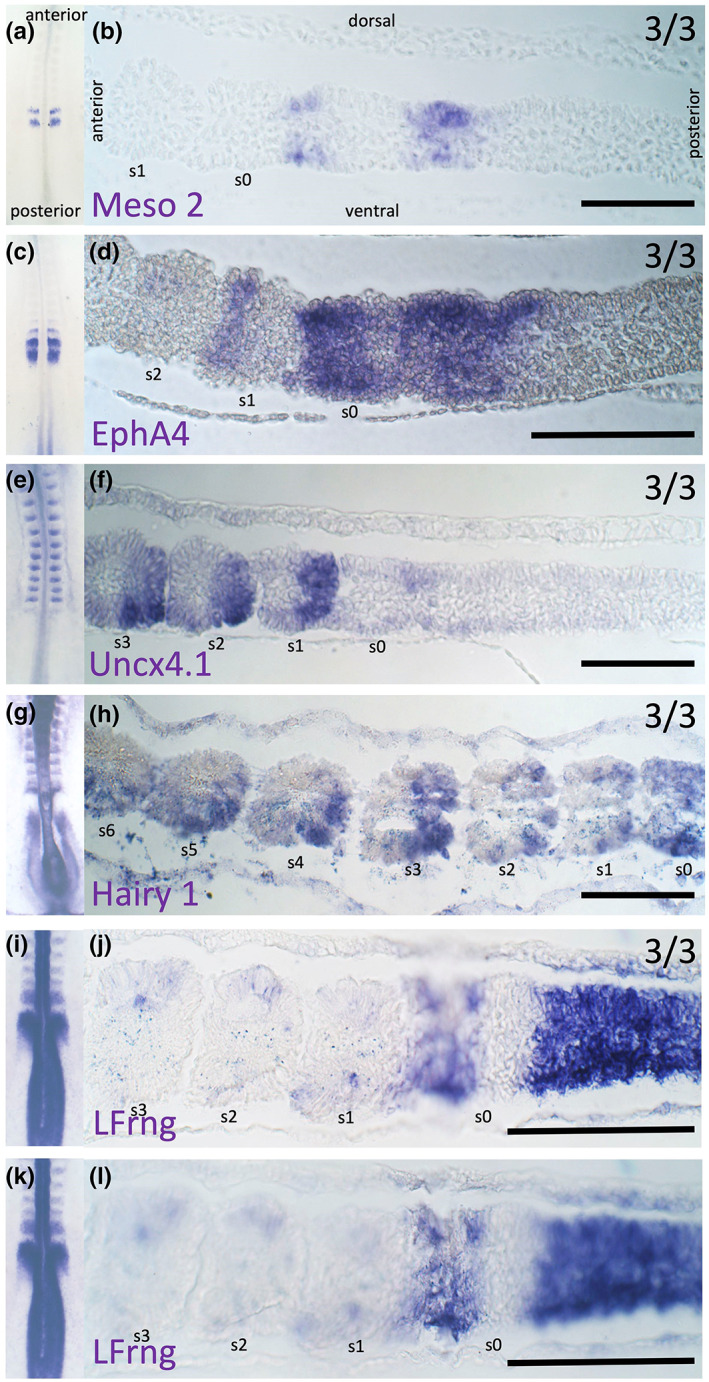
Expression of the rostral and caudal somite markers. (a) Whole‐mount in situ hybridization for Meso 2. (b) The same embryo sectioned sagittally. (c) Whole mount of EphA4 and (d) sagittal section. (e) Whole mount of Uncx4.1 and (f) sagittal section. (g) Whole mount of Hairy1 and (h) sagittal section. (i, k) Whole mount of LFrng; (j and l) show the same sagittal section imaged at different focal planes. Scale bars are 100 μm. Three embryos per marker were analysed. S‐somite.

Because core cells never epithelialize, we asked whether they behave differently from other somite mesoderm cells in other aspects of their cellular dynamics, for example whether they have rostro‐caudal somite identity. The rostral‐somite marker *EphA4* (Baker & Antin, 2003; Gilardi‐Hebenstreit et al., 1992; Schmidt et al., 2001) and caudal‐somite markers *Meso2* (Buchberger et al., 2002), *Uncx4.1* (Schrägle et al., 2004), *LFrng* (McGrew et al., 1998) and *Hairy‐1* (Palmeirim et al., 1997) markers were examined by in situ hybridization to determine whether the mesenchymal cells in the core have rostro‐caudal identities (Figure 7). All these markers have quite dynamic expression in the PSM and only become confined to the rostral or caudal domains in the forming newly formed somite, therefore their value as rostral or caudal markers is restricted to already epithelialized somites. After epithelialization, the rosette morphology of the somite allows identification of core cells with greater confidence.

*Meso2* is expressed in the anterior PSM as 1–3 narrow bands (Buchberger et al., 2002). Embryos with two bands were selected for further analysis (Figure 7a,b). The most anterior stripe is localized in the posterior part of the forming somite (s0): dorsal and ventral cells show clear expression, but core cells show either very low or no expression (Figure 7b). In the second stripe (slightly more posterior in the PSM), dorsal cells express *Meso2* more strongly than other parts of the PSM (Figure 7b).

In newly formed (s1) somites, *EphA4* is expressed in the anterior domain perhaps including some core cells (Figure 7c,d). In the forming somite (s0) expression of *EphA4* is visible in dorsal, ventral and anterior domains but the posterior and the posterior‐core domains are unstained (Figure 7d).

*Uncx4.1* is a robust caudal marker, which starts to be expressed as soon as the somites separate from the PSM (Figure 7f). Somites s1‐s3 have strong expression in their posterior domain, and weaker expression in a subset of core cells neighbouring the posterior domain (this observation contradicts a previous report of *Uncx4.1* expression being restricted to the somitocoele (Schrägle et al., 2004)).

*Hairy1* and *LFrng* expression oscillates strongly in the PSM but both transcripts become confined to the caudal somite as soon as it separates from the PSM (McGrew et al., 1998; Palmeirim et al., 1997) (Figure 7g–l). In s1‐s3, *Hairy1* is expressed only in the posterior domain; most core cells do not appear to express it (Figure 7h). Expression of *LFrng* becomes localized to the neighbouring parts of two adjacent somites: the anterior domain of somite s0 and the posterior domain of somite s1 (Figure 7j,l). More rostral somites have weak expression of *LFrng* in the dorsal and ventral domains (Figure 7j). Most of the core cells of somites s0‐s3 do not express *LFrng* (Figure 7j,l).

**FIGURE 8:**
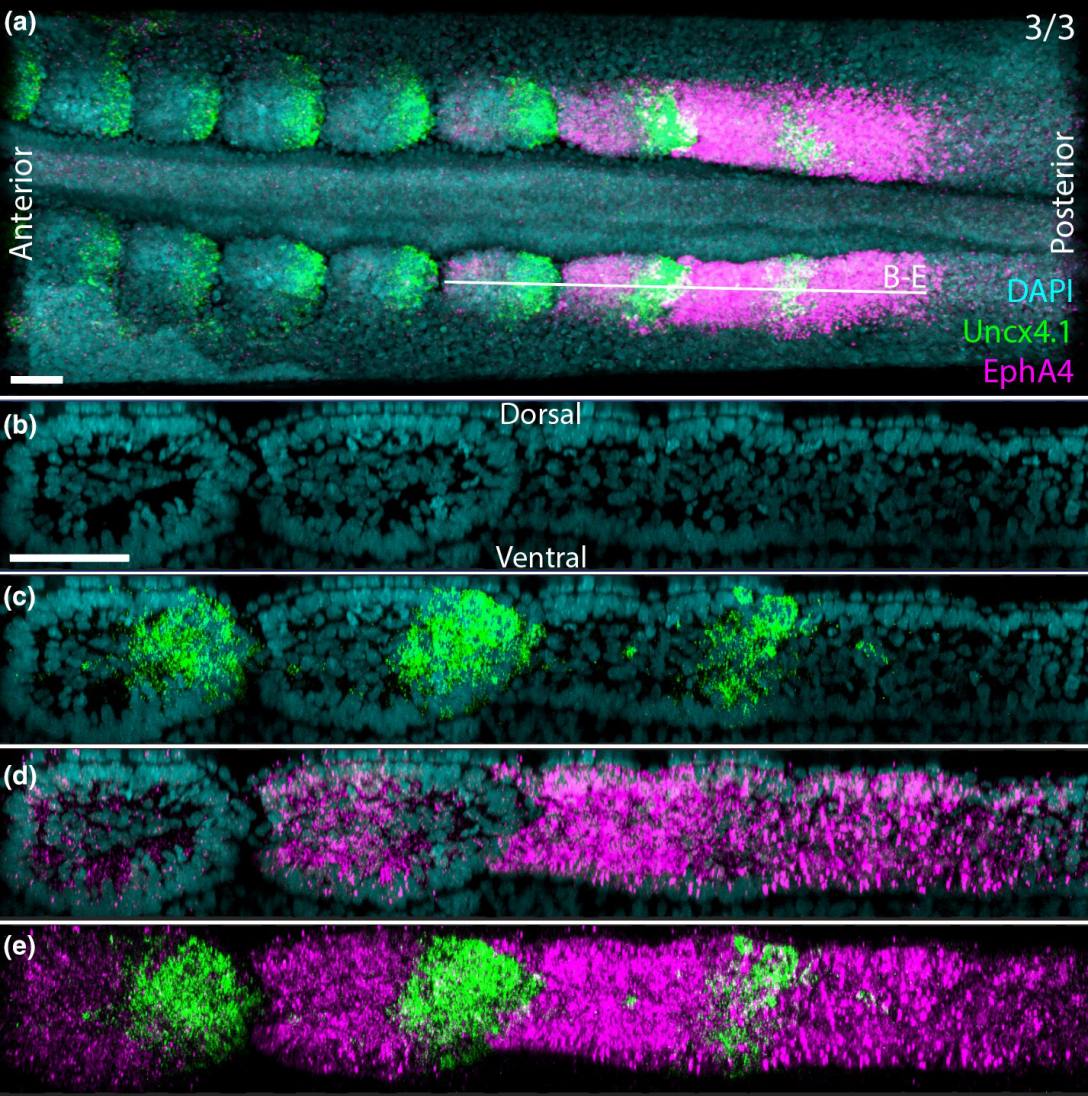
Multiplexed expression of rostral and caudal somite markers. (a) Whole‐mount hybridization chain reaction (HCR) for Uncx4.1 and EphA4 shown as a 3D maximum projection in the coronal plane (b–e) Sagittal sections (5 μm thickness) of the same embryo generated computationally showing DAPI (b), DAPI and Uncx4.1 merged (c) DAPI and EphA4 merged (d) Uncx4.1 and EphA4 merged (e). Scale bars are 50 μm. Supplementary Movie S3 provides a three‐dimensional view of the staining pattern reconstructed from the *x*‐*y*‐*z* confocal image stack.

In summary, within the forming and newly formed somites: *Hairy1* and *LFrng* posterior expression does not extend to the core of the somites, *EphA4* expression is restricted to some anterior core cells, Meso2 is absent from the core cells of somites and *Uncx4.1* is detectable in a subset of posterior core cells. The observation that only a subset of the core cells in each half‐somite seems to express the markers could indicate either that core cells are not as robustly specified as rostral or caudal as the somite epithelium, or that substantial cell mixing continues within the core after somite formation. To distinguish between these hypotheses, hybridization chain reaction (HCR) (Dirks & Pierce, 2004) was used to visualize transcripts for *EphA4* and *Uncx4.1* simultaneously (Figure 8). In formed somites s1‐s2, where both markers are expressed (Figure 8a), the caudal marker *Uncx4.1* clearly extends to the core (Figure 8a,c,e), as does the rostral marker *EphA4* (Figure 8a,d,e), as viewed in coronal and sagittal sections. However, a few cells expressing each marker are seen within the other marker's territory of expression. In 3‐d reconstructions of the virtual confocal image stack (Supplementary Movie S3), the territory of *EphA4* expression is larger than the caudal domain (*Uncx4.1*). Moreover, the boundary between rostral and caudal halves of the core, as revealed by these markers, appears somewhat oblique, with *EphA4* being expressed more strongly in the anteromedial aspect of the somite and *Uncx4.1* showing the reverse orientation. Therefore, in newly formed somites, there is indeed a boundary that extends to the core, but this is not precisely orthogonal to the axis of the embryo. This observation suggests that von Ebner's fissure, which does appear to separate exact somite halves (Stern & Keynes, 1987), may not exactly coincide with the boundary of expression of these markers. This suggests that cells adjust their expression a little later, or that cell mixing occurs at this stage, or that some cells may be eliminated. It has been reported that some core cells can incorporate into the epithelial wall of the somites (Huang et al., 1994, 1996; Kulesa et al., 2007; Kulesa & Fraser, 2002; Martins et al., 2009), which may contribute to tidying up this boundary.

In contrast to the pattern in formed somites, the domain of expression of *EphA4* in the PSM appears much larger than the prospective rostral half (Figure 8a,d,e). A few cells apparently co‐express both markers. Again, the change between the domains of expression before and just after segmentation could suggest cell sorting or changes in expression. In conclusion, our results suggest that the rostral‐caudal subdivision of the somite extends into the core, although this boundary may not correspond precisely to that defined by von Ebner's fissure.

## 4 DISCUSSION

### 4.1 Order of cellular events leading to somitogenesis

A previous study reported that the posterior two‐thirds of the PSM is mesenchymal and that the first signs of epithelialization are observed in the anterior PSM (Duband et al., 1987). The dorsal PSM appears to epithelialize more posteriorly (earlier) than the ventral PSM (Bellairs, 1979) and the medial PSM before the lateral PSM (Kulesa & Fraser, 2002; Martins et al., 2009). The present SEM study suggests that epithelialization begins at a position 40% of the length of the PSM (from the caudal end) for dorsal PSM cells, then medially at the 55% position, and then at the same level for ventral and lateral domains at 70% PSM. Thus, the first signs of epithelialization (40% PSM length) appear much more posteriorly (earlier) than previously reported (Bellairs, 1963; Bellairs, 1979; Duband et al., 1987; Kulesa & Fraser, 2002; Martins et al., 2009). Sagittal and transverse SEM sections also clarify the order of events between the dorsal and medial PSM. In contrast to previous reports of somitomeres spanning at least half the length of the PSM (7–9 prospective somites) (Meier, 1984), we could only observe ‘pre‐somite’‐like cell arrangements for, at most, 1–3 presumptive somites in the most anterior‐PSM.

*Paraxis* (*TCF‐15*) is considered a good marker for the onset of epithelialization as it is first expressed in the anterior PSM (and retained in formed somites). *Paraxis* mutants generate somite‐sized arrangements of cells but these do not have epithelial structure (Burgess et al., 1995, 1996; Kulesa et al., 2007). However, the present results reveal that the first signs of epithelialization are seen at 40% position in the dorsal PSM, well before *paraxis* is expressed.

Epithelialization seems to generate discrete epithelialized clusters in the dorsal and then medial PSM (Figures 1h and 2d,f). This raises the question of what regulates the appearance and spacing of these clusters. Previous studies showed that ectoderm is required for epithelialization and, together with the neural tube, is required for *paraxis* expression, which is consistent with the appearance of the clusters in the dorsal and medial aspects of the PSM (Correia & Conlon, 2000; Lash & Yamada, 1986; Packard Jr, 1976; Sosic et al., 1997). However, the PSM was also shown to epithelialize autonomously, provided that the fibronectin matrix is intact (Lash & Yamada, 1986; Rifes et al., 2007).

3D‐confocal analysis of polarity markers PKCζ_,_ PAR3, ZO1, Golgi marker GM130 and apical marker N‐cadherin revealed that each marker starts to be localized at a characteristic position and domain of the PSM: this pattern is different for each marker as well as from the pattern of cell elongation described above (Figure 4 and Supplementary Table S8). This observation suggests that epithelialization and polarization may be regulated independently of each other.

### 4.2 Are the observed PSM cellular dynamics compatible with existing models of somite formation?

To date, none of the models can quite explain all the experimental observations. For example, the ‘clock and wavefront’ model is challenged by the finding that a single heat‐shock can generate repeated (periodic) defects in segmentation, whereas the model predicts only one anomaly should occur (Cooke, 1978; Elsdale et al., 1976; Keynes & Stern, 1988; Primmett et al., 1988, 1989; Roy et al., 1999). In addition, somites can be generated from posterior primitive streak, which normally does not form paraxial mesoderm; these somites form almost simultaneously, are not arranged along a line, and lack oscillatory expression of the ‘clock’ genes of the Notch pathway, yet have approximately normal size and shape. Therefore, they appear to form without participation of either the clock or a wavefront (Dias et al., 2014; Streit & Stern, 1999). In silico simulation predicts that somites can be generated spontaneously if cells are allowed to have neighbours, can ‘see’ both the apical and the basal sides of those neighbours, and can maximize their adhesion and epithelialize once they come into close contact with each other (Dias et al., 2014). Hence, studying cellular PSM dynamics in vivo is of interest.

The ‘clock and wavefront’ model proposes that a group of PSM cells undergo ‘*rapid cell change’* at the same time, as a sudden ‘catastrophic’ change (Cooke & Zeeman, 1976). Thus, the model predicts that a group of PSM cells cluster together in synchrony, rapidly, and at the same time as they commit to form a somite together. In this study, analysis of the sequence of cell shape and polarization changes revealed that the PSM is subdivided into domains that undergo the changes separately, and for each domain, the change is gradual (except for lateral PKCζ, Figure 4), begins at a different PSM level, and occurs at a different time and rate—in other words, the timing of events does not seem to be characteristic for a particular somite. These observations seem to contradict the idea that the clock‐and‐wavefront functions primarily to determine the size of somites (by regulating the number of cells that will later segment together) and the timing of segmentation. However, they do not exclude the possibility that the ‘rapid cell change*’* may be manifested in other cellular behaviours not studied here, such as cell adhesion, or simply the ‘commitment’ to undergo these behaviours. The finding that the core domain never epithelializes is also not obviously consistent with the ‘clock and wavefront’ model, but may be accommodated by invoking that core cells may lack ‘competence’ to respond to the clock and wavefront information.

The ‘cell cycle’ model proposes that the cells within the PSM are organized according to their age order and their cell cycle phase, and that some cell‐cycle‐coupled event taking place prior to segmentation is responsible for gating cells into cohorts that will segment together (Collier et al., 2000; Primmett et al., 1989; Stern et al., 1988). Although the original proposal was that this gating event might take place one cell cycle (about 10 h) before overt segmentation, it could also take place earlier. This would be more consistent with the finding that groups of cells are seen to start epithelialization as early as the 40% position in the PSM (which is a little longer than 10 h prior to overt somite formation). However, since epithelialization events start at different times/positions for each domain of the PSM, this would suggest that the interpretation of the gating information may differ for each property and position. As with the clock‐and‐wavefront model, the failure of core cells to participate in epithelialization could be explained by lack of competence of these cells to interpret the information.

The ‘reaction diffusion’ model proposes a posterior‐to‐anterior morphogen gradient to which cells respond while oscillating between two cellular states (Meinhardt, 1982). In the context of epithelialization dynamics, the two cellular states could be epithelial and mesenchymal, one of which becomes fixed after a cell experiences a certain level of the morphogen. Because neighbouring cells promote the opposing state, this scenario is compatible with epithelialized clusters observed at the dorsal‐posterior‐PSM, but not in the dorsal‐anterior‐PSM where all cells are already epithelialized. As with the cell cycle model, because the epithelialization pattern is domain specific, the model would also require separate, and different, interpretations of this information at each position/domain, including the core.

The PORD model (Cotterell et al., 2015) proposes that cellular interactions at local level induce the neighbouring cells to change their state, and that this change propagates posteriorly. The epithelialization pattern within the PSM fits this concept as the epithelialization is more advanced in the anterior than posterior PSM for most of the domains. As for other models, the PORD model does not predict that core cells never epithelialize, that each PSM domain epithelializes at a different PSM level, or that epithelialized clusters appear at posterior‐dorsal‐ and posterior‐medial‐PSM levels, which may require differences in interpretation of the information.

The ‘wave and polarization’ model stemmed from observations of epithelialization dynamics within the PSM (Beloussov & Naumidi, 1983; Polezhaev, 1992). In the dorsal‐PSM, epithelialized fans of cells recruit neighbouring cells to form a somite. The model proposes that ‘primary polarized cells’ appear within the PSM as a consequence of the interplay between the cell cycle and a wave of ‘somitogenic cell determination’. The epithelialized cell clusters at dorsal and medial‐posterior‐PSM levels could be equivalent to Polezhaev's primary polarized cells. Again, this model does not explain why different PSM domains epithelialize at different rates, timing and position within the PSM, or why the core never epithelializes.

Our initial observations that dorsal‐PSM forms an epithelial monolayer long before somite formation led to a model being proposed in which an interplay between apical constriction and a posteriorly progressing ‘activation front’ alone could segment the dorsal‐PSM (Adhyapok et al., 2021). The model predicts that segment size increases as the speed of an ‘activation front’ increases, and that there is an inverse rate of increase for apical contractility. However, the behaviour of other PSM domains was not integrated into this model—perhaps, separate activation fronts operate in each domain, but it is then unclear what is responsible for the formation of each somite, involving all the surfaces of the PSM (and not the core).

In conclusion, none of the existing models can easily explain our present findings. At the very least, differences in the interpretation of the segmentation information are required between different groups of cells to account for differences in the timing of segmentation in the different domains of the PSM, and the failure of the core to participate in segmentation.

### 4.3 Sequence of epithelialization events within the somites

Epithelialization within the newly forming somite (s0) is a gradual process, with its anterior domain reportedly epithelializing before its posterior domain (Duband et al., 1987; Kulesa et al., 2007; Kulesa & Fraser, 2002) or, conversely, its posterior domain preceding its anterior domain (Beloussov & Naumidi, 1983; Martins et al., 2009). The work presented here uncovered no significant difference in AR between the anterior and the posterior domains within forming and formed somites, suggesting that both domains may epithelialize together (Figure 3 and Supplementary Table S5). Epithelialization within the newly formed somite was reported to coincide with separation from the PSM, with the separated somite having a rosette structure (Kulesa & Fraser, 2002). However, the present results show different epithelialization dynamics. When a somite separates from the PSM its anterior and posterior domains are still mesenchymal; cells in the dorsal and ventral domains are elongated to a different extent, and then cells in each domain separately, at a different somite age, reach their maximum elongation to form a regular rosette (Figure 3, Supplementary Table S5). This may be a consequence of epithelialization order within the PSM.

The core cells of the somite never epithelialize (Figure 3 Supplementary Tables S5 and S6). However, Beloussov and Naumidi proposed that the ‘cell fan’ of the posterior‐somite domain is generated when the epithelialized dorsal domain recruits core cells, which then elongate. The ‘fan’ was said to progress in a dorsal‐to‐ventral direction (Beloussov & Naumidi, 1983), but in Figure 1e‐f, the ‘fan’ is also visible in the ventral domain of the forming somite. It is not certain whether there is recruitment of the core cells or an inward movement of the dorsal and ventral epithelium towards each other.

The anterior domain of somite 1 has fewer elongated cells than the posterior domain of somite 2 (Supplementary Table S4, Figure 3a,b). This suggests that the border between neighbouring somites may form due to different degrees of epithelialization between neighbouring somites, a mechanism previously proposed for the separation of somites from the PSM (Beloussov & Naumidi, 1983). Structures similar to the ‘cell fans’ are observed between somite 1 or 0 and the anterior front of the PSM (Figure 3a,b). Cells within this region were not allocated to anterior‐core‐PSM, and were not analysed. However, Beloussov and Naumidi's proposed that the somitic border forms as a consequence of epithelialization differences are challenged by the *paraxis* mouse null mutant which undergoes segmentation but does not epithelialize, suggesting that epithelialization is dispensable for segmentation. Moreover, their model does not provide a mechanism for the regulation of somite size or the timing of segmentation.

## 5 THE CORE CELLS

The core cells, or somitocoele, are characteristic for amniotes like mouse and chick but are not a feature of anamniotes like zebrafish or frog, as the cellular organization of the somite is different in these taxa. In chick, core cells occupy the centre of a somite, but can relocate to the epithelial rosette, as established by time‐lapse observation of living embryos (Kulesa et al., 2007; Kulesa & Fraser, 2002; Martins et al., 2009) as well as fate mapping of core cells (Huang et al., 1994, 1996). Core cells have been reported to arise by proliferation and ingression from the epithelialized rosette (Martins et al., 2009; Wong et al., 1993). Here, the core cells seem to be recruited from the centre/middle cells of the unsegmented PSM, which are engulfed by the elongating cells of the surrounding domains. This observation raises the question as to whether core cells are merely an architectural leftover, or whether there is a mechanism regulating formation and number of the core cells, as well as a specific molecular identity and polarity.

The core cells of the PSM behave differently from the cells of other domains. Our results suggest that the core population never epithelializes (Figure 1m). Also, although polarization was not directly studied within the core domain, the core cells do not seem to polarize, as no increased immunostaining for polarity markers is observed within this domain (Supplementary Figures S3–S12). Since our observations are based on fixed embryos, they do not allow us to establish whether there is shuttling between the core cells and other domains at the PSM level. Indeed, this possibility cannot be excluded as other studies have suggested extensive cell mixing at posterior‐PSM levels (Benazeraf et al., 2010, 2017; Selleck & Stern, 1991; Stern et al., 1988). Our findings with HCR to reveal expression of a caudal (*Uncx4.1*) and a rostral (*EphA4*) marker in the same embryo show that the boundary between these markers changes over time. Prior to segmentation, the domains of expression of *EphA4* are larger than expected for a somite half; as the somite forms, the domain of expression of *Uncx4.1* becomes larger than that of *EphA4*. Moreover, the boundary between the two domains in the somite is not precisely orthogonal to the embryo's long axis. These observations are consistent either with cell sorting or with dynamic changes in gene expression.

Shuttling and the suggestion that core cell fate is not irreversibly determined (Martins et al., 2009; Senthinathan et al., 2012) could explain the versatile fate of the core cells, which contribute to myotome, sclerotome and its derivatives, ribs, and the annulus fibrosus of the intervertebral discs, as well as intervertebral joints (Huang et al., 1994, 1996; Mestres & Hinrichsen, 1976; Mittapalli et al., 2005; Williams, 1910). Shuttling could also explain disagreements about the origin of the annulus fibrosus which has been reported to be derived from rostral and caudal sclerotomes, as well as from the core cells (Bruggeman et al., 2012; Huang et al., 1994; Takahashi et al., 2013).

A related question is whether core cells have medio‐lateral identity. cSim1 (lateral) and cSwiP (medial) markers are expressed in the core cells of newly formed somites (Pourquie et al., 1996; Vasiliauskas et al., 1999), suggesting that they do have medio‐lateral identity. This medial‐lateral boundary appears to be much sharper than the rostral‐caudal boundary within the core.

## CONFLICT OF INTEREST

The authors declare no conflicts of interest for this paper.

## Supporting information


Data S1

Movie S1

Movie S2

Movie S3

## Data Availability

All original data are available from the authors on request.
