# Supplementary material for: Sequential changes in cellular properties accompanying amniote somite formation
Source: J Anat. 2022 Nov 24;242(3):417–35. doi: 10.1111/joa.13791 (PMC9919497; doi:10.1111/joa.13791)
Supplement: Supplementary file 1 — Data S1 [file JOA-242-417-s004.pdf]

Supplementary Methods

1 PSM length re-scaling

Regardless of whether the embryos had formed the same number of somites at a given stage, there are embryo-to-embryo variations in the total length of the PSM due to variability of the size of the entire embryo. To compensate for this and for the fact that embryos at around stage HH 11 (1) (11-15 somites) were used, the total length of the PSM was converted into a percentage value. Before any cut was made the total length of the PSM was measured using a lens reticule, counting from the newly formed somitic border between s1 and the PSM (referred to here as 100% of the PSM length) to the chordo-neural hinge (CNH) (this location is referred to here as 0% of the PSM length). After the transverse cut was made the distance from the newly formed somite border to the plane of the cut was also measured and calculated according to the formula: cut position / total length of PSM, and this was represented as a percentage. In sagittal PSM fractures the most posterior portion of the PSM was determined based on the morphology of the endoderm and ectoderm, which begin to approach one another, and is referred to as 0% PSM. This anatomical feature corresponds to the place where the neural tube ends, the CNH. The sagittal fractures also revealed the somites during their formation. Because of the dynamic nature of somite formation some embryos have a s0 and some do not. If cells were assigned to s0 they were not classified as PSM, and the cells behind s0 were considered as the most anterior PSM, referred to as 100% PSM. Otherwise, 100% PSM falls between s1 and the PSM border.

2 Assigning cells to PSM or somite domains

For sagittal PSM fractures the cells were assigned to dorsal, ventral or core domains. For transverse fractures of the PSM the cells were classified into five domains: dorsal, ventral, medial, lateral and core. For sagittal fractures cells of the newly formed somites and s0 were classified into anterior, posterior, dorsal, ventral and core domains. Classification was made manually using the following criteria: a given cell can belong to only one domain. All domains, except the core, consist of a single cell layer that is the outermost part of the PSM or of the somites. The cells of the dorsal

domain are those that lie closest to the ectoderm; those neighbouring the neural tube and/or notochord belong to the medial domain. Cells adjacent to endoderm fall into the ventral domain, and those neighbouring the lateral mesoderm belong to the lateral domain. Cells adjacent to two structures (e.g., neural tube and ectoderm) were assigned to the domain based on which structure was in greater contact with that given cell. Cells that did not neighbour any structure but were enclosed by other PSM cells were classified as being in the core (somite lumen). The anterior and posterior domains of the sagittal section of the somites were determined based on the anteriorposterior orientation of the embryo. They form a single celled layer and are not adjacent to either the ectoderm or endoderm. Cells in the most anterior part of the PSM that are not adjacent to the ectoderm or endoderm were classified as core.

3 SEM analysis and statistics

3.1 Regression methodology for SEM images of sagittal fractures through PSM

In the case of sagittal section images, regression analysis was used for two purposes: first, to model the upper surface of the embryo in order to straighten it mathematically, and second, to model the variation in aspect ratio (AR) along the line of the sagittal fracture. The cell-level data for all embryos was arranged in a single table, with seven columns: embryo id, cell id, X and Y coordinates of the centre of mass of the cell, its aspect ratio (AR), cell region (PSM/somite), and cell type (dorsal/ventral/core etc.). Processing of the embryos led to shrinkage and therefore some distortion, which was particularly evident in sagittal sections. The surface of the embryo was no longer flat. Correction of this curvature was the first step in data processing. An algorithm to straighten the upper surface of the embryo was applied as follows:

1. Fit a curve roughly following the upper surface. To do so, the entire embryo was split into 30 sections. In each section, the uppermost cell's coordinates were taken. These 30 X-Y coordinates provide the data to fit a curve to the upper surface. The number 30 was chosen heuristically based on inspection of the curvature of the embryos and the resolution (number of cells) available.
2. If the image of a straight embryo was simply rotated by an angle, the relationship between X and Y would be linear, while for curved surfaces higher order

polynomials would be necessary to model it. There were also instances where there was a dip or rise in the middle of the embryo, which is modelled more accurately by a trigonometric curve such as sine or cosine. Therefore, to flexibly model the curve, the first two components of a polynomial basis (X and X^2) were taken, along with two components from a Fourier basis (sin and cos, at two periods).

3. When using a trigonometric curve, its period (λ_1) is an important parameter. By trial-and-error, two periods were selected: $\lambda_1 = 1$ and $\lambda_1 = 1.5$
4. The fitted regression line was therefore:

$$Y \sim \alpha + \beta_1 \cdot X + \beta_2 \cdot X^2 + \beta_3 \cdot \sin(X \cdot \lambda_1) + \beta_4 \cdot \sin(X \cdot \lambda_2) + \beta_5 \cdot \cos(X \cdot \lambda_1) + \beta_6 \cdot \cos(X \cdot \lambda_2)$$

5. This equation was fitted for the 30 X-Y coordinates corresponding to the upper surface. This gives a mathematical representation approximately following the upper surface. The resulting models were plotted on the embryo images to check for consistency visually.
6. Subsequently, for any cell, using its X value, we estimated where the upper surface would be, by putting the X value in the equation. That gave us the predicted Y value, $\hat{Y}$.
7. Upon subtracting the predicted Y value from the actual Y value, we get the estimated Y value $Y^* = Y - \hat{Y}$ which would occur if the upper surface was perfectly horizontal at Y=0.
8. All subsequent analysis is performed with this straightened (warped) value, Y^*.

Because embryos vary slightly in length before further processing, the data were scaled horizontally along the X axis, so that the tail end of the PSM was at 0%, and the PSM-somite boundary was at 100%. Cells in the somite therefore had X values greater than 100%, while cells in PSM had values between 0-100%.

A first level of analysis was performed to study how cell aspect ratio changes along the PSM and as this becomes partitioned into somites, for the various domains (dorsal, ventral, and core). For all analyses, data were amalgamated across all embryos.

To model how the aspect ratio of cells changes along the PSM, it was observed that the relationship between AR and distance (*d*) was non-linear, and therefore a sigmoid (S-shaped) curve was fitted, for each of the three domains in the PSM. The equation of this curve was:

$$AR \sim \theta \cdot \mathrm{sigmoid}(\alpha \cdot (d - \beta)) + \gamma$$

Where γ is the intercept (constant), θ is the slope (steepness) of the curve, β is the centre (midpoint) of the curve, and α the scaling factor (representing how distance affects the shape of the curve).

The sigmoid curve is very flexible, as it can approximate linear or quadratic/exponential models according to its parameters. When α is very large and β is near the centre of the PSM range (i.e., 50%), it is close to a linear curve. When β is beyond the PSM range (i.e., $\beta > 100\%$), it is similar to a quadratic/exponential curve. When $\alpha = 0$, *d* has no effect, and it is a horizontal straight line. When α is very small, the curve can also resemble a stair i.e. represent a sharp change.

A wide choice of sigmoid functions is available as implemented in statistical packages, and their performances are similar. Here, the cumulative distribution function (CDF) of the standard normal distribution (Gaussian error function), was used, as it has good mathematical properties, such as symmetry around the midpoint of the curve along both X and Y axes.

Most analyses were done in R version 3.3.2, using MASS for statistical analysis, and ggplot2 for figures. MATLAB version R2017a was used to fit the sigmoid curve and to generate the histograms.

3.2 Regression methodology for SEM images of transverse fractures through somites

The cell-level data for all embryos was arranged in a single table, with seven columns: sample id, cell id, distance (scaled distance from tail, 0-100%), aspect ratio (AR), side (left/right), and cell type (dorsal, ventral, medial, lateral and core).

Transverse sections were obtained only at specific points for each embryo, so distance in this case was not continuous from the tail end, as was the case for sagittal sections. It was only possible to obtain measurements at specific distances, in this case: 0, 10, 40, 45, 55, 60, 65, 70, 90, 95, and 100%. For exploratory analysis, box plots were made with AR as the dependent variable, and distance and cell type as grouping variables. Left and right sides were grouped together for all analyses. Because the distance was not continuous in this case, to compare domains for regression analysis, a simple linear regression model was assumed, as it would be difficult to estimate parameters accurately for higher order regression models.

Regression analysis was performed by relating AR to distance and domain (coded as indicator variables). Interaction terms between domain and distance were also used, to allow domains to have different rates of increase. These were confirmed by performing regression analysis within a single domain, where interaction terms are not necessary.

To test whether there are significant differences between PSM domains along the PSM, a t-test was performed. Since there are 5 domains, ${}^{5}C_{2}$ = 10 tests were done in total. To adjust for multiple testing, the Bonferroni corrected threshold for significance is 0.05/10 = 0.005.

3.3 Regression methodology for SEM images of sagittal fractures through somites

First, a boxplot for AR was made for each somite and each domain within each somite. All somite cells across all embryos were aggregated for the plot. This plot indicates the change in epithelialization in each domain as somites continue to form.

To test if there were significant differences between each domain of a given somite Wilcoxon test was used to calculate P-values. For each somite, ${}^{5}C_{2}$ = 10 tests were done, so 6×10 = 60 tests were done in total. To adjust for multiple testing, the Bonferroni corrected threshold for significance is $0.05/60 = 8.33\times10^{-4}$.

To test if there is a significant difference between a given domain and somite number, a comparison was done using regression analysis of AR against somite number, with P-values obtained from a t-test (Bonferroni corrected significance threshold 0.05/5 = 0.01).

To test if there are significant differences for each domain between consecutive somites, a pairwise comparison was done using the Wilcoxon test (5×5 = 25 tests, so Bonferroni adjusted significance threshold = 0.05/25 = 0.002).

To see how AR changes between the anterior and posterior borders of neighbouring somites all cells that fall in the anterior domain of one somite were compared to all cells falling in the posterior domain of the next somite. The AR values were compared using a two-sided t-test, with the p-values and total number of cells analysed presented in the results. These results were plotted by showing P-values against somite number. As five comparisons were made in total, the Bonferroni adjusted significance threshold is 0.05/5 = 0.01, shown as the red horizontal line in the plot.

4 Mounting for whole mount immunostaining

Due to the requirement for long scanning times under the confocal microscope and resulting quenching of the TOPRO nuclear stain, embryos were either stored in the secondary antibody or individually processed as follows. Embryos were individually transferred to a depression slide and positioned with their ventral side uppermost. The embryo was then washed several times in PBS. Those embryos that had been fixed in 1% PFA at 4°C for 15 min were very fragile; to reinforce them they were post-fixed in 4% PFA for a further 15min in the dark. Then, the embryo, still on the depression slide, was carefully dehydrated with isopropanol in PBS in 10%, 20%, 30%, 40%, 50%, 60%, 70%, 80%, 90%, 3x 100% series. Next, the embryo was cleared and mounted in benzyl alcohol: benzyl benzoate (BABB) (1:2). Because this clearing agent makes the embryo invisible, fluorescence (blue incident light and green bandpass filters, using the Nightsea system in a dissection microscope) were used to reveal the embryo more easily. A coverslip was applied, sealed with superglue and secured with parafilm

for further protection as BABB can dissolve superglue. In uncleared embryos only 1/3 of a somite depth could be scanned.

5 3D confocal imaging

3D images of fixed and immuno-stained embryos were acquired with an Olympus FV1000 inverted microscope equipped with a 30x silicon oil immersion objective (N.A.=1.05, working distance W.D.=0.8mm) with a coverslip correction collar set from 17mm to infinity. The slides were placed in a rotating insert, which enabled precise embryo positioning and consequent reduction of the number of tiles needed to cover the region of interest. The embryos were imaged from the ventral side. Silicon immersion oil (Olympus) of refractive index RI=1.406 was used as the closest available match to BABB mounting medium (RI =1.559). To scan the region including the PSM and newly formed somites, 12-16 tiles with 5% overlap and 1 µm step size were taken with a 1.5x digital zoom, each with 1024 x 1024 resolution and using 2x Kalman averaging.

A single image took 5-9 hours to acquire and was 10-14 GB in size. Next, the individual tiles were stitched together one by one in FIJI using “3D stitching” (Plugins>Stitching>deprecated>3D stitching) plugin (Preibisch et al., 2009). To speed up the stitching process a small region of overlap was manually selected so the plugin was not applied to the entire tile. All other available stitching plugins, or bulk stitching, resulted in misalignments and distortions of the overlapping regions. There was some obvious loss of signal at overlapping regions, which could not be prevented either by the use of DABCO anti-quenching agent or by lowering the laser intensity. These losses were due to photobleaching as increasing the size of the region of overlap by 20% resulted in a corresponding increase of the faded field. After acquisition, there was a visible grid on the sample, corresponding to the tiles. To minimise this, 5% overlap was selected, which was the minimum required to obtain good quality stitching without losing signal from a more significant area.

6 Confocal image processing

The acquired 3D stacks were processed for further analysis in FIJI (Fig. S13). The images were checked to ensure that they retained their original pixel dimensions of

0.276 length x 0.276 height x 1 depth as stitching changed it to 1x1x1. Because there are no obvious anatomical cues to determine the boundary between the lateral-PSM and lateral mesoderm, especially at the level of the posterior-PSM, this boundary was defined by trimming off the lateral and posterior tissues (Fig. S13 B). The tissues were cropped manually by drawing a line from the lateral border of the somites and the lateral mesoderm, and then the line was run parallel to the neural tube finally to curve behind the CNH region (which was determined in XZ orthogonal slices). Next, the trimmed 3D images were transversely re-sliced every 10 pixels with the ‘reslice’ command in FIJI (Stacks>Reslice) (Fig. S13 C).

Next, the resulting X, Z transverse image sequences were saved as separate Tagged Image File Formats (TIFF) for each channel. Slices that contain the intersomitic space, or the anterior or posterior edges of a somite, or the very anterior end of the PSM, were not used for further analysis.

7 Determination of zones for analysis

All embryos stained for polarity markers were processed in the same way. Three embryos each for GM130, PKC[illegible], PAR3, and NCad with TOPRO nuclear stain and 2 embryos for ZO1 with TOPRO were processed. To analyse the pattern of polarity markers within the PSM and newly formed somites the following steps were followed. First, the embryos had their PSM length calibrated, as the same stage embryos vary in PSM length. The posterior part of the PSM was represented as 0% and its anterior tip as 100% corresponding to the most recently formed (most caudal) somite border (Fig. S14 A). The 0% position was placed where there is no obvious morphological distinction between PSM, notochord and LM, and where the neural tube is fused to the caudal end of the notochord. This region corresponds to the CNH. Next, four domains (dorsal, ventral, medial and lateral) were determined and halved to be further subdivided into apical and basal zones. This defines 8 zones in total. The core was not considered for this analysis as there are no apical and basal zones within the somitocoele, and some of the basal zone can be located in the core and thus bias the result. Then, by measuring the intensity difference of a given polarity marker between

the apical and the basal zones of a given domain, the change in cell polarization along the PSM could be determined.

Determining the four domains was challenging as the outlines of individual cells cannot be distinguished within the mass of PSM and somites, mainly because BABB clearing replaces all the lipids of the cell membrane and renders the cells completely translucent. To overcome the limitations, the following strategy was developed to estimate the dimensions of PSM cells at all axial levels. To define the four domains, outlines of the somites (Fig. S14 C) and PSM (Fig. S14 D) were drawn on left and right side of an embryo by using the TOPRO channel. Using the TOPRO channel instead of a channel with a polarity marker prevented human bias. The same FIJI tools were used as for SEM analysis. By increasing brightness and contrast of the TOPRO channel it was possible to estimate the outlines of axial tissues based on the nuclei position and manually draw lines along them. Next, the outlines were saved as individual regions of interest (ROI) in ROI manager in FIJI. Then, four points were drawn in the most dorsomedial, mediolateral, ventrolateral and ventromedial parts of somites (Fig. S14 E, yellow crosses) and PSM (Fig. S14 F, yellow crosses) to define the limits of the dorsal, ventral, medial and lateral domains. Then, in MATLAB, virtual lines were drawn from each of those points to the centre of mass of the outlines (Fig. S14 E-F red dots are centres of masses), to subdivide the ROI into the four domains. The ROI with the outlines can be transferred from the channel with the TOPRO nuclear stain to the channel with a polarity marker, in this case PKC□ (Fig. S14 G, H). Next, the depth of each domain was determined by assuming that a given domain has a depth equal to the length of a single cell (Fig. S14 B). This assumption is based on the observation that a somite is a rosette, with the outer layer a single cell in depth. The lengths of the mesenchymal cells in the posterior-PSM and the epithelial somitic cells were manually measured by using GFP-mosaic embryos as a reference. These had been electroporated with a GFP plasmid construct and imaged in the same way as the experimental embryos.

The mean length of the mesenchymal posterior-PSM cells was 10 □m and the mean length of an epithelialized somite cell was 19.79 □m. The cell length is thus less in the

posterior-PSM than at somite level. In addition, the SEM images revealed that the AR of cells was different in the different domains at the same axial level, and cells of the PSM do not elongate in the same linear manner at different levels of the PSM. The rate of change in AR was calculated in the SEM analysis by fitting a curve to the AR value and then plotted this value against distance along the PSM.

Embryos used for confocal analysis underwent some shrinkage during preparation. The average outline area (e.g., Fig. S14 C-D, yellow lines) at somite and posteriorPSM levels was calculated in the mosaic-GFP reference embryos. The outline area for the experimental embryos was also calculated at somite and 0% PSM levels, to estimate a “shrinkage factor”. The amount of shrinkage in the experimental embryo can vary along the embryo. At somite levels (>100% PSM), the shrinkage factor is the ratio of somite area in an experimental embryo divided by average somite area in reference embryos. The same was done with measurements in the posterior-PSM (0% PSM). Finally, the shrinkage factor was linearly interpolated between 0% and 100% of the PSM. The calculated distances from the manually drawn outlines to the centre of mass were multiplied by this shrinkage factor. The outlines of each domain were then contracted by this factor to obtain the inner boundary for that domain, which is the boundary between that domain and the core (Fig. S14 I, J dashed lines). By contracting the outer boundary by half of the estimated length of a cell, the midboundary of each domain was obtained (Fig. S14 I, J dotted line). This was used to split the domain into basal (outer; solid line) and apical (inner; dashed line) zones (Fig.
S14 I, J).

8 Intensity calculations and regression analysis

Once the domain boundaries were determined, which split the somites and the PSM into four domains with basal and apical zones each, the average intensities could be calculated for each zone. For each embryo, average intensities were obtained for each X Z slice, for the left and right sides, and for basal/apical zones of each domain. To determine how cell polarity as revealed by a given marker changes for dorsal, ventral,

medial and lateral domains along the PSM, the basal intensities were subtracted from the apical intensities. Next, data points from all X Z slices per marker, per domain, were aggregated. The position of a slice (calibrated within each embryo, so that 0 = beginning of PSM, 100 = PSM / somite boundary, and > 100 for somites) was taken as the independent variable (X) and the apical-basal intensity difference as the dependent variable (Y). A regression of the intensity difference (Y) was performed against position (X). A plot of Y against X indicated that a sigmoid function most appropriately could be used to fit these data (also similar to what was described above for SEM data analysis):

$$Y \sim \theta \cdot \mathrm{sigmoid}(\alpha \cdot (X - \beta)) + \gamma$$

The sigmoid function was fitted separately for each domain and each marker. In each case, the four parameters $\theta, \gamma, \alpha, \beta$ were estimated from the data to calculate the best fitting sigmoid curve, and an F-test was used to test whether the model fit was significant. For any sigmoid curve, the inflection point is the point (X value) along the curve at which its rate of increase (i.e., slope of the curve at that point) is highest. This can be calculated as:

$$I_1 = \beta - \frac{\sqrt{1.5}}{\alpha}$$

If the sigmoid curve spans the entire range of the data, then the Y value at this inflection point I_1 is approximately 11% of the range of the data (Y). However, depending on the data, the inflection point in a particular case can be outside the observed range of the data. Therefore, to ease interpretation, a second value I_2 was calculated, which is 10% of the observed range of the data (Y).

$$I_2 = min(Y) + 0.1 \times [max(Y) - min(Y)]$$

Inflection points I_1 and I_2 indicate the position along the X axis at which the Y value starts to increase more rapidly. To see how well the sigmoid curve fits the data, correlations between the original data and the fitted values were calculated.

9 Live imaging and time vs distance calibrations

For extended culture time-lapse imaging, in vitro cultures were set in special chambers as described (2). Embryos were labelled with DiI (Life Technologies, C7001) (1:10 in 0.03M sucrose and 1:10.000 Tween-20 in distilled water), caudal to the CNH. Next, an individual embryo was filmed for 26-34h at 10 min intervals for red and bright fields with a Zeiss Axioskop upright microscope, equipped with 5x dry objective (NA 0.15), an air curtain incubator set to 38°C, motorised stage, and a Hamamatsu C4742-95 digital camera controlled using HCI image software (Hamamatsu). A Prior Lumen 2000 lamp with fibre optics guide and a set of chroma filters were used for epifluorescence.

To translate the distance of the PSM into time before cells become incorporated into a somite, the distance between the leading labelled cells and the most recently formed somite border was measured. The total amount of growth of somite tissues was estimated by measuring the distance between the posterior PSM and the first border formed since the beginning of acquisition. The PSM length was then subtracted from the gap between the labelling and the newly formed somite to obtain the relative time needed to form a somite (Fig. 5A, 6A).

References

1. Hamburger V, Hamilton HL. A series of normal stages in the development of the chick embryo. J Morphol. 1951;88(1):49-92.
2. Kucera P, Burnand MB. Routine teratogenicity test that uses chick embryos in vitro. Teratog Carcinog Mutagen. 1987;7(5):427-47.

Table S1. Number of cells per domain per embryo in sagittal fractures

Number of embryos, with somites (s) formed	Number of cells per domain		
	Dorsal	Ventral	Core
1. s11	171	122	660
2. s11	159	83	556
1. s12	158	104	595
1. s13	169	113	457
2. s13	173	127	508
1. s14	153	91	325
2. s14	119	88	282
3. s14	130	80	485
4. s14	131	87	466
5. s14	125	94	396
6. s14	149	47	428
1. s15	126	73	288
Total	**1763**	**1109**	**5446**

Table S2. Number of cells per distance per domain in transverse SEM fractions

PSM distance [%]	Number of embryos	Number of cells per domain				
		Dorsal	Ventral	Medial	Lateral	Core
0	3	67	52	39	34	197
10	1	15	7	12	9	54
40	1	14	12	13	8	19
45	3	105	55	36	25	200
55	1	14	9	10	11	41
60	1	12	12	11	10	37
65	1	30	18	14	4	28
70	3	80	73	55	29	199
90	3	75	59	51	14	139
95	1	32	27	21	4	65
100	2	34	26	20	18	12

Table S3. Variable comparisons of transverse SEM fractures

Domain	Lateral	Ventral	Medial	Dorsal
Core	9.46×10^{-3}	2.47×10^{-8}	2.89×10^{-15}	5.38×10^{-71}
Lateral		0.167	1.21×10^{-3}	6.66×10^{-18}
Ventral			0.020	3.67×10^{-20}
Medial				8.75×10^{-10}

Table S4. Number of cells per domain in all sagittally fractured somites

Somite no	Number of samples with this somite available in sagittally fractured sample	Total number of cells per domain across all embryos				
		Core	Anterior	Posterior	Ventral	Dorsal
s0	7	126	45	47	68	89
s1	11	117	107	128	129	126
s2	5	64	60	65	70	69
s3	5	60	80	58	74	75
s4	4	55	37	70	66	66
s5	3	42	45	45	53	44

Table S5. Domain comparisons within a somite

Somite 0	Posterior	Dorsal	Ventral	Core
Anterior	0.35	3.19×10^{-10}	0.0005	0.26
Posterior		1.09×10^{-8}	0.0062	0.03
Dorsal			0.0005	1.03×10^{-18}
Ventral				8.64×10^{-8}
**Somite 1**				
Anterior	0.74	4.11E-11	0.0002	9.11×10^{-11}
Posterior		3.36E-12	2.7×10^{-5}	1.10×10^{-10}
Dorsal			0.0036	9.19×10^{-28}
Ventral				2.60×10^{-21}
**Somite 2**				
Anterior	0.84	7.94E-04	0.11	8.3×10^{-11}
Posterior		7.43E-05	0.08	1.41×10^{-13}
Dorsal			0.04	4.92×10^{-19}
Ventral				2.48×10^{-16}
**Somite 3**				
Anterior	0.42	2.65×10^{-6}	0.14	1.08×10^{-14}
Posterior		2.89×10^{-4}	0.53	8.88×10^{-15}
Dorsal			0.0012	2.38×10^{-20}
Ventral				8.79×10^{-17}
**Somite 4**				
Anterior	0.83	1.12×10^{-4}	0.0012	2.65×10^{-11}
Posterior		8.74×10^{-7}	2.38×10^{-5}	4.68×10^{-16}
Dorsal			0.32	1.80×10^{-20}
Ventral				1.61×10^{-18}
**Somite 5**				
Anterior	0.0162	3.80×10^{-4}	0.98	$1.9x10^{-14}$
Posterior		1.14×10^{-7}	0.04	$2.83x10^{-11}$
Dorsal			0.0005	$2.77x10^{15}$
Ventral				$3.46x10^{14}$

Table S6. Comparisons between domain and somite number.

Domain	P-value
Anterior	1.54×10^{-19}
Posterior	3.59×10^{-19}
Dorsal	1.63×10^{-19}
Ventral	6.03×10^{-19}
Core	0.9967

Table S7. Comparisons of the same domain for consecutive somites

Domain	Somite comparison				
	0-1	1-2	2-3	3-4	4-5
Anterior	0.026	0.000431	0.060	0.564	0.003
Posterior	2.56×10^{-1}	2.59×10^{-6}	0.009	0.776	0.273
Dorsal	0.521	0.002	0.007	0.389	0.017
Ventral	0.133	0.002	0.090	0.007	0.831
Core	0.999	0.515	0.256	0.748	0.606

Table S8. Time readouts for epithelialization and polarisation events

Domain	Event	Inflection point (X)	Embryo 1 Time [hh:mm]	Embryo 2 Time [hh:mm]	10% height point (X)	Embryo 1 Time [hh:mm]	Embryo 2 Time [hh:mm]
Dorsal	GM130	75.52	26:20	22:50	74.73	26:20	22:50
	PAR3	83.18	29:10	26:40	82.17	29:10	25:10
	PKCζ	44.08	17:10	18:00	43.12	17:10	16:30
	ZO1	82.79	29:10	25:10	71.90	26:20	22:50
	Ncad	64.27	23:40	20:50	60.43	22:00	20:50
	AR	44.80	17:10	16:30	42.77	15:10	16:30
Ventral	GM130	71.94	26:20	22:50	62.42	23:40	20:50
	PAR3	119.37	>34:10	>26:40	56.30	22:00	18:00
	PKCζ	55.92	17:10	18:00	49.96	19:50	18:00
	ZO1	110.92	>34:10	>26:40	103.85	>34:10	>26:40
	Ncad	91.53	29:10	25:10	90.75	32:10	25:10
	AR	70.26	23:40	20:50	66.02	23:40	20:50
Medial	GM130	71.20	23:40	20:50	60.64	22:00	20:50
	PAR3	147.49	>34:10	>26:40	71.67	26:20	22:50
	PKCζ	83.97	26:20	22:50	67.47	23:40	20:50
	ZO1	103.45	>34:10	>26:40	91.51	32:10	26:40
	Ncad	93.16	32:10	26:40	86.90	29:10	25:10
	AR	N/A	N/A	N/A	N/A	N/A	N/A
Lateral	GM130	93.23	32:10	26:40	92.72	32:10	26:40
	PAR3	116.68	>34:10	>26:40	49.84	19:50	18:00
	PKCζ	100.00	34:10	26:40	100.00	34:10	26:40
	ZO1	98.30	34:10	26:40	98.01	34:10	26:40
	Ncad	92.09	32:10	26:40	91.82	32:10	25:10

	AR	N/A	N/A	N/A	N/A	N/A	N/A
**Core**	AR	N/A	N/A	N/A	N/A	N/A	N/A

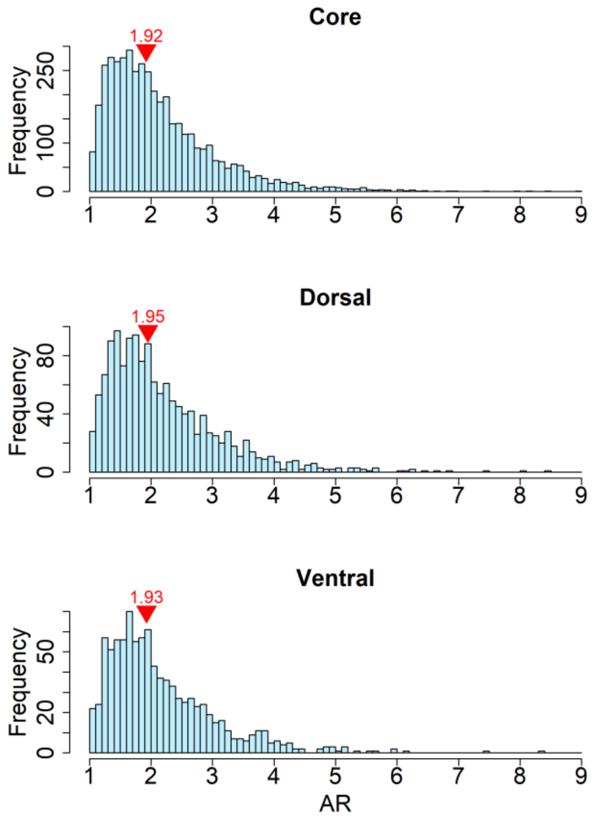

Fig S1. AR frequency (cell number) distribution per domain of cells within sagittal PSM sections. The majority of cells in each domain have a low AR of 1-3 with the core having the highest frequency (300) compared to dorsal (100) and ventral (60) domains. Cells in the core reach an AR of 6.5, whereas dorsal and ventral cells have an AR of 8.5. Red arrows indicate the median.

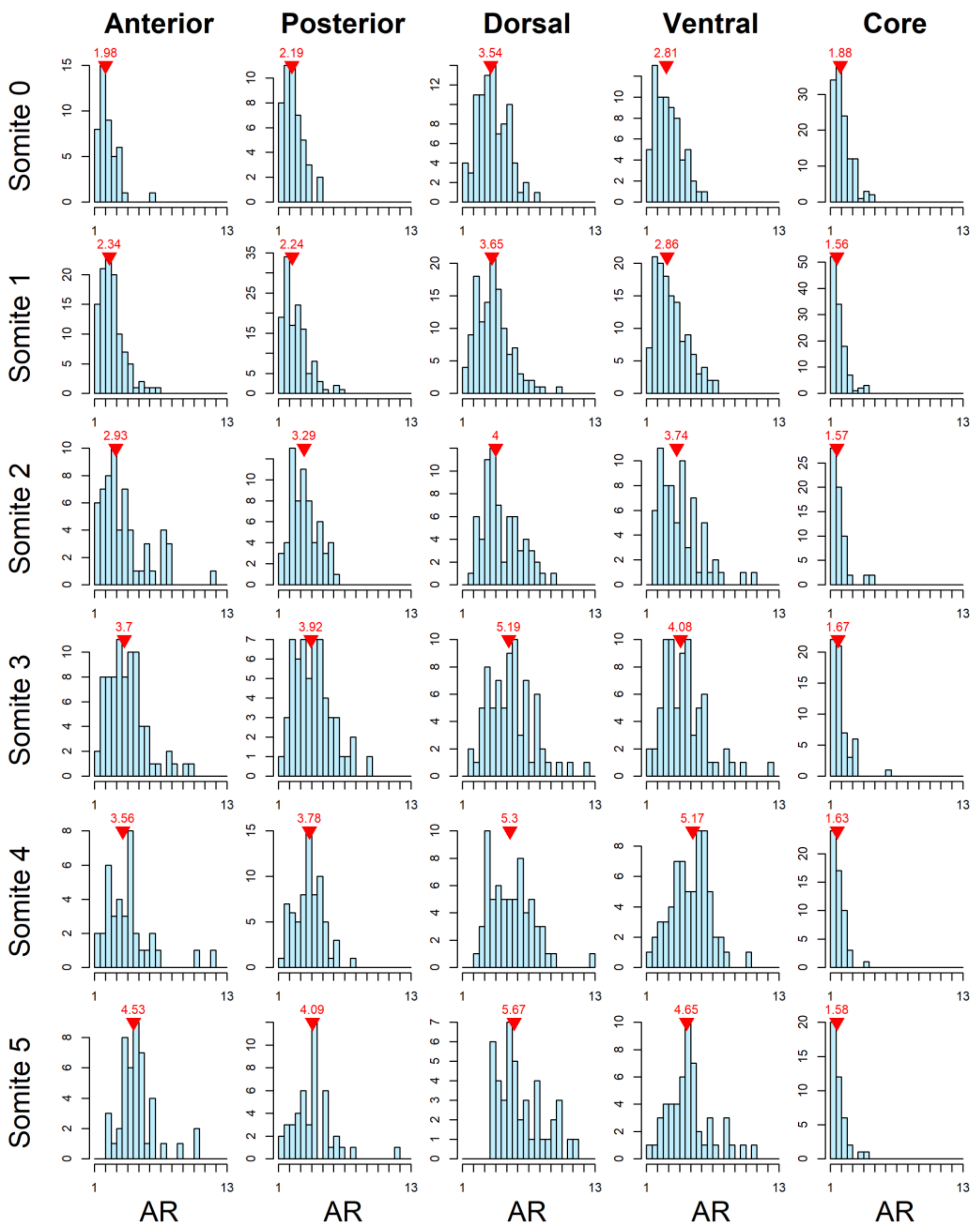

Fig S2. AR frequency (number of cells) distribution per domain per somite. The majority of core cells in all somites have low AR, as histograms are shifted to the left. These cells are mostly mesenchymal. The majority of cells of the ventral and dorsal domains in all somites have high AR, as the histograms have a bell-shaped distribution. These cells are mostly epithelial with occasional mesenchymal cells. The histograms of the anterior and the posterior domains shift from the left (somite 0-1) to the right (somite 2-5), which suggests that the anterior and posterior cells of a given somite epithelialize together and are more elongated in older somites. The red arrows point medians.

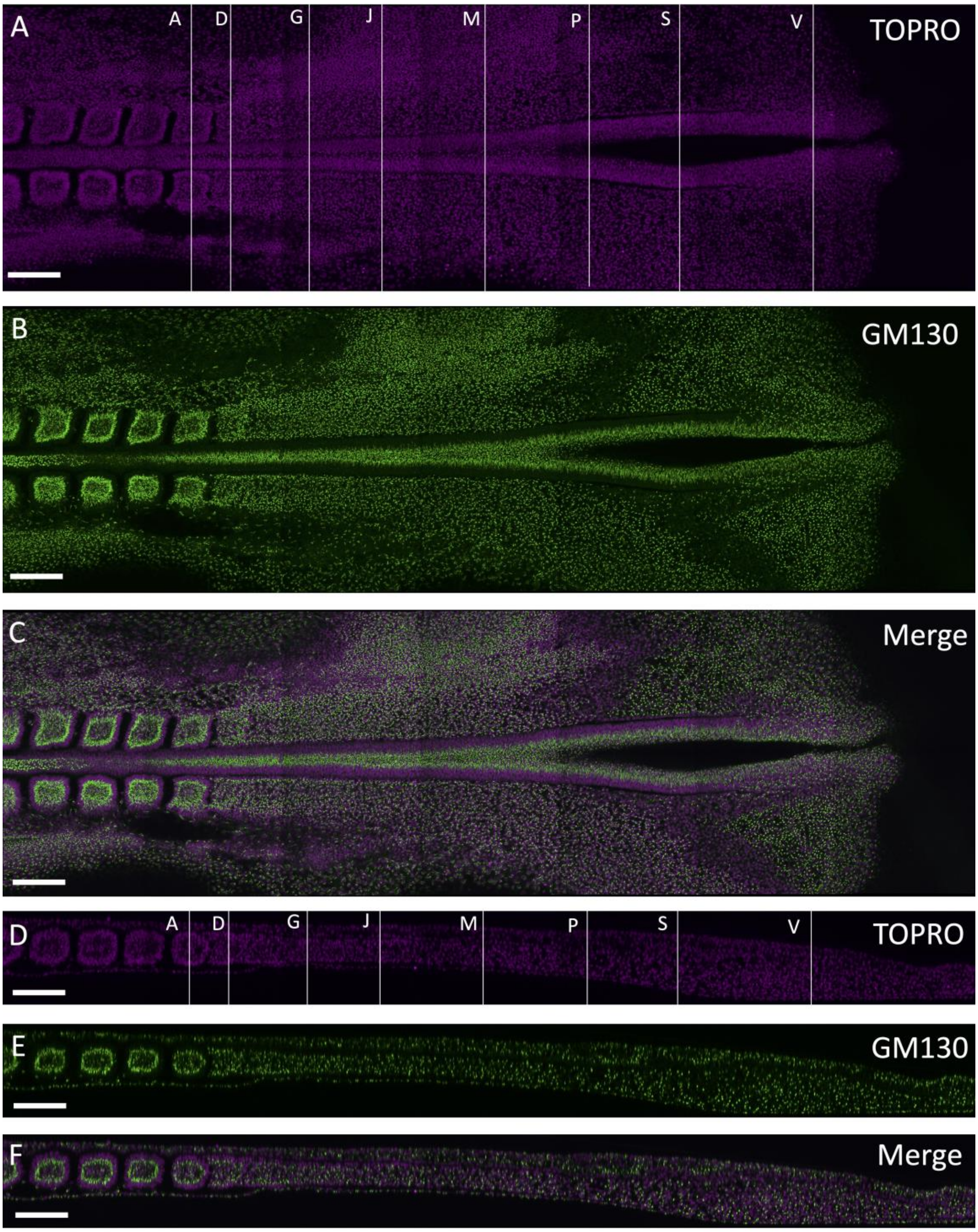

Fig S3. 3D localization of GM130 and TOPRO, coronal and sagittal planes. A-C) Coronal planes (x, y). D-F) Sagittal planes (y, z). The white bars (A-V) in A and D correspond to the axial levels of transverse planes (x,z) presented in Fig S4. Scale bars are 100 μm. Anterior to the left.

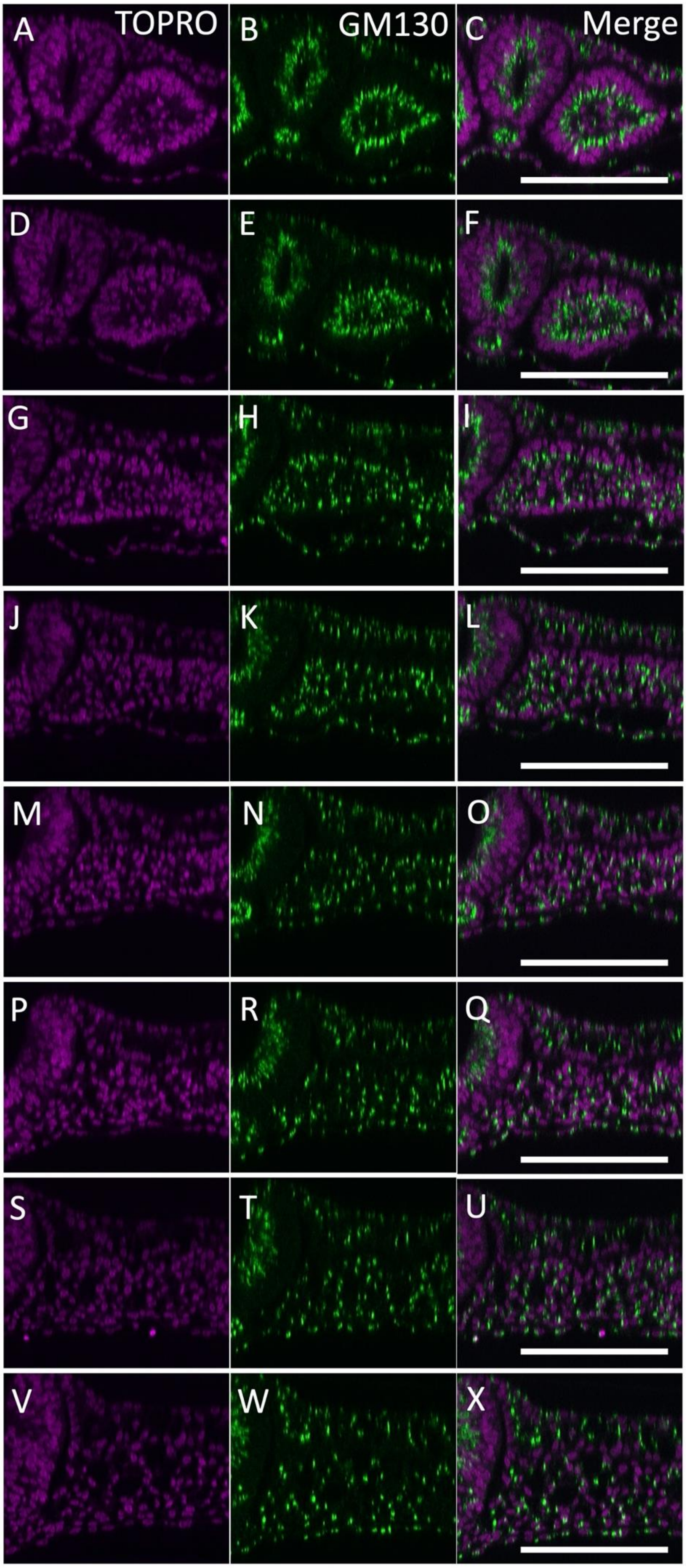

Fig S4. 3D localization of GM130 and TOPRO, transverse planes. A-X) Transverse planes (x, z) at different axial levels as indicated in Fig S3 A, D (white bars). Dorsal to the top. Scale bars are 100μm.

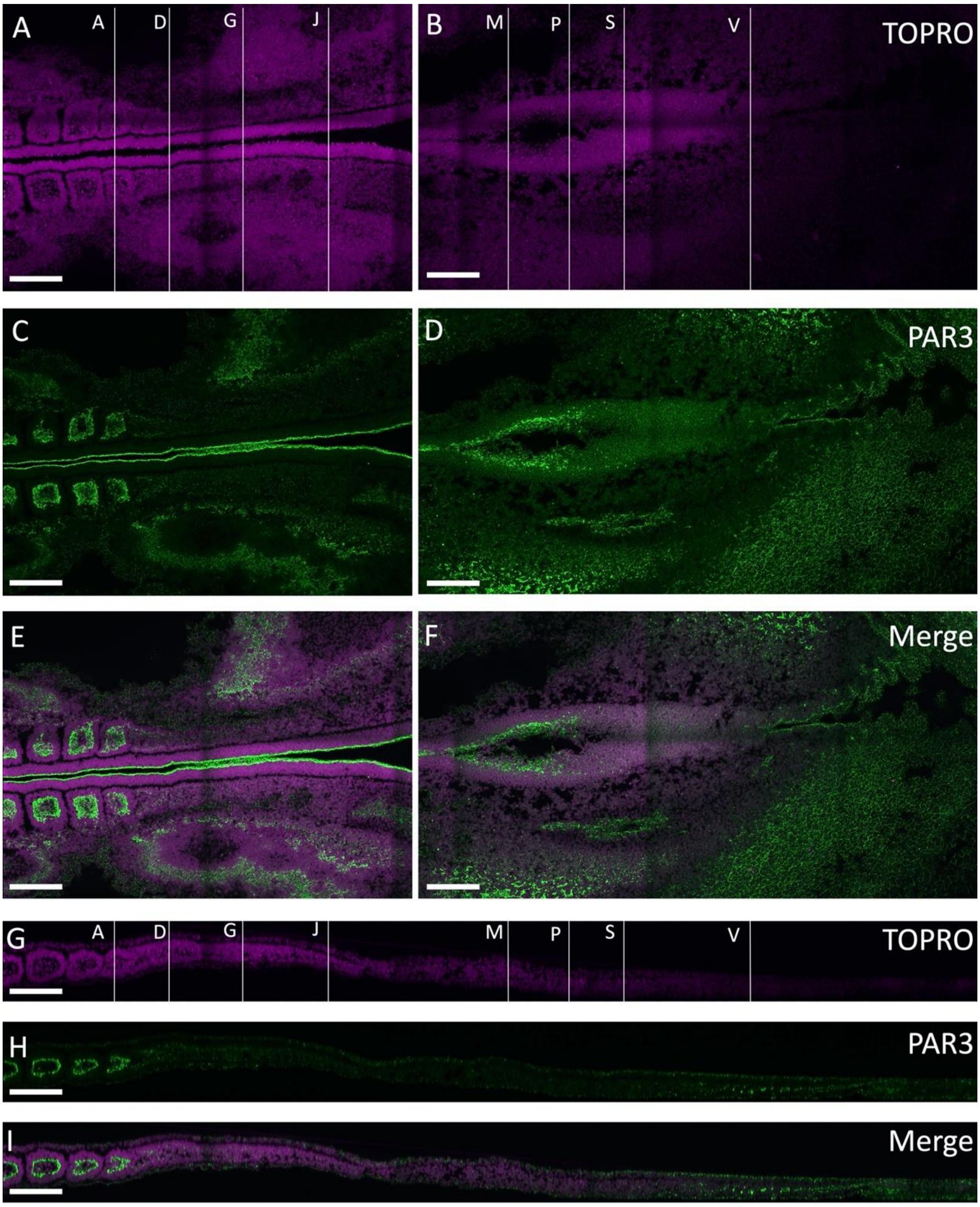

Fig S5. 3D localization of PAR3 and TOPRO, coronal and sagittal planes. A-F) Coronal planes (x,y). B, D, F show a different x,y plane than A, C, E as the embryo is bent. G-I) Sagittal plane (y,z). The white bars (A-V) in A, B, G correspond to the axial levels of transverse planes (x,z) presented in Fig S6. Scale bars are 100 μm. Anterior to the left.

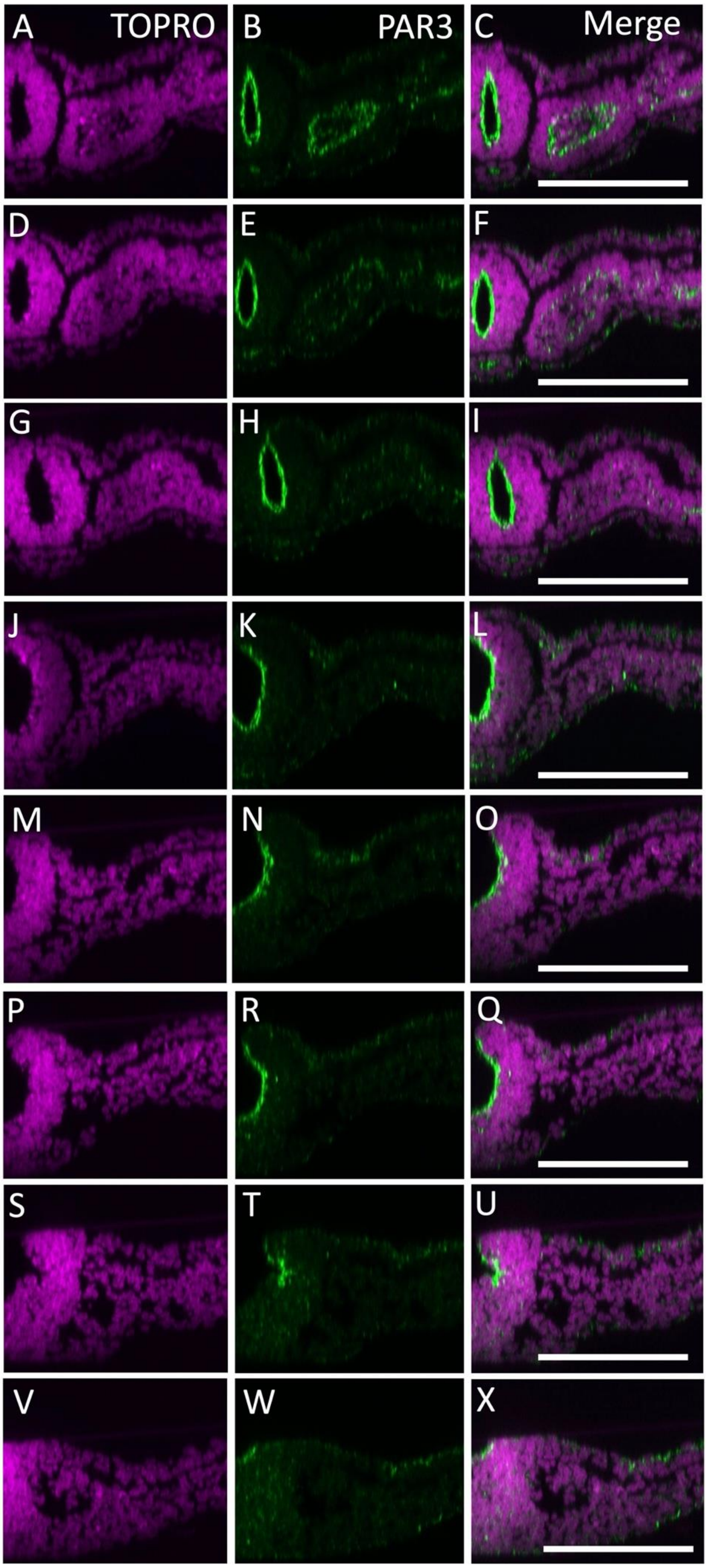

Fig S6. 3D localization of PAR3 and TOPRO, transverse planes. A-X) Transverse planes (x,z) at different axial levels as indicated in Fig S5. A, B, G (white bars). Dorsal to the top. Scale bars are 100μm.

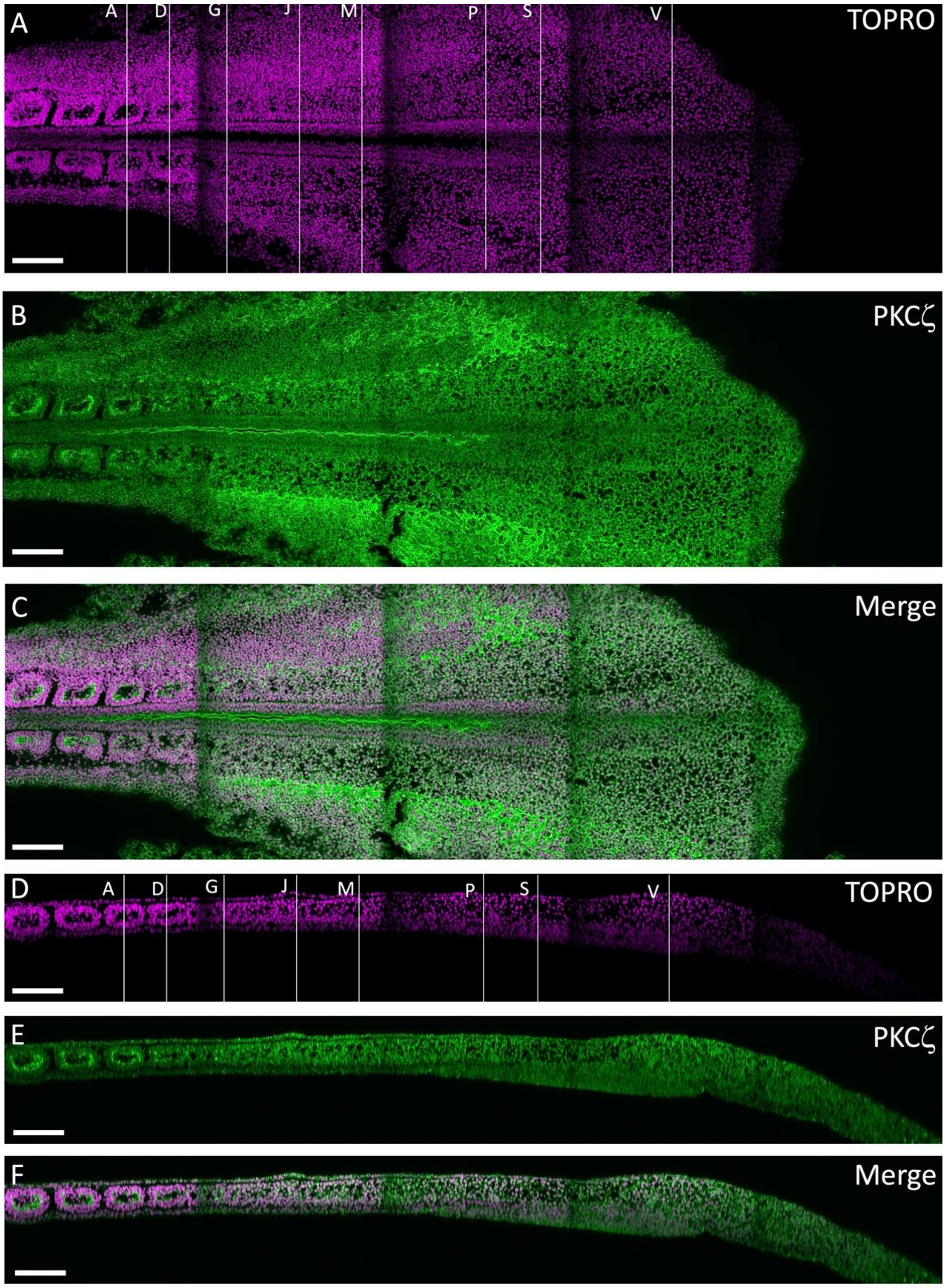

Fig S7. 3D localization of PKCζ and TOPRO, coronal and sagittal planes . A-C) Coronal planes (x,y). D-F) Sagittal planes (y,z). The white bars (A-V) in A and D correspond to the axial levels of transverse planes (x,z) presented in Fig S8. Scale bars are 100 μm. Anterior to the left.

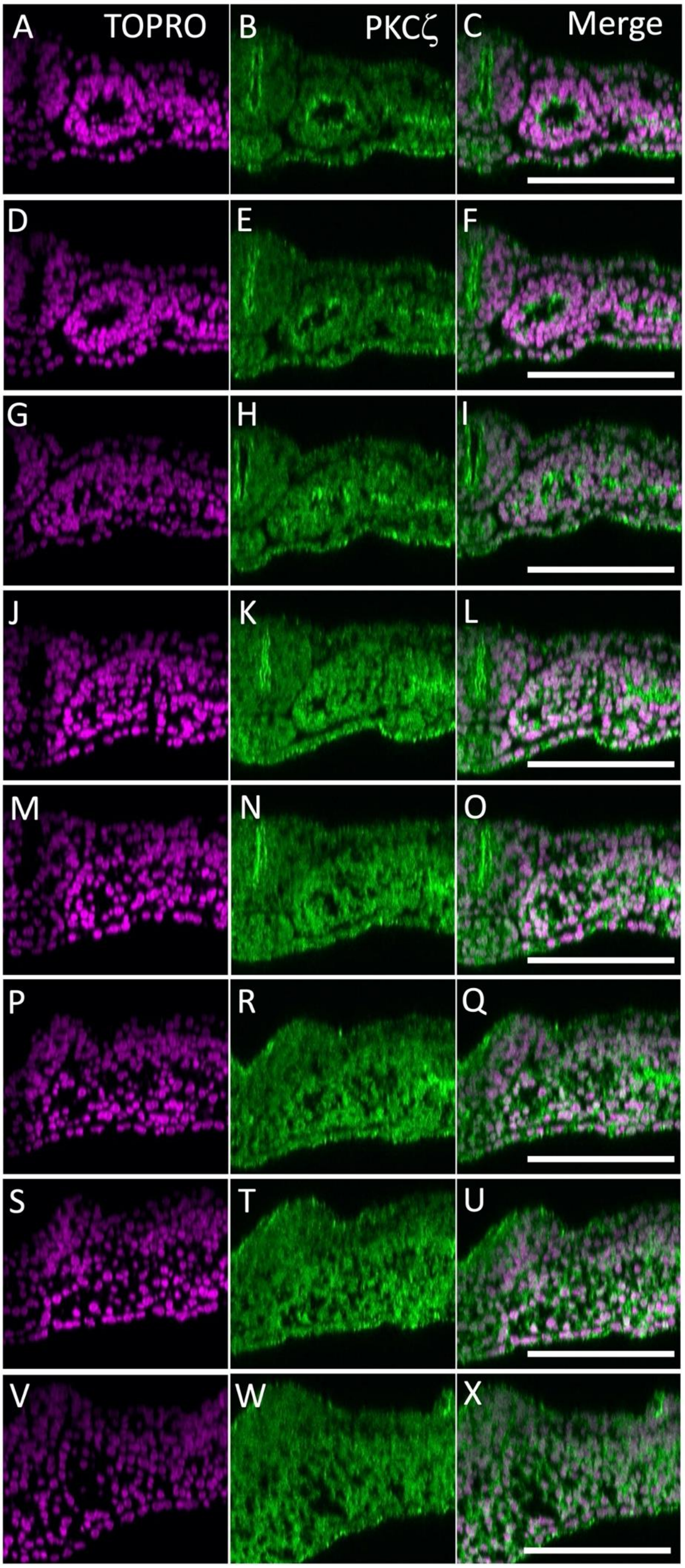

Fig S8. 3D localization of PKCζ and TOPRO, transverse planes. A-X) Transverse planes (x,z) at different axial levels as indicated in Fig S7 A , D (white bars). Dorsal to the top. Scale bars are 100µm.

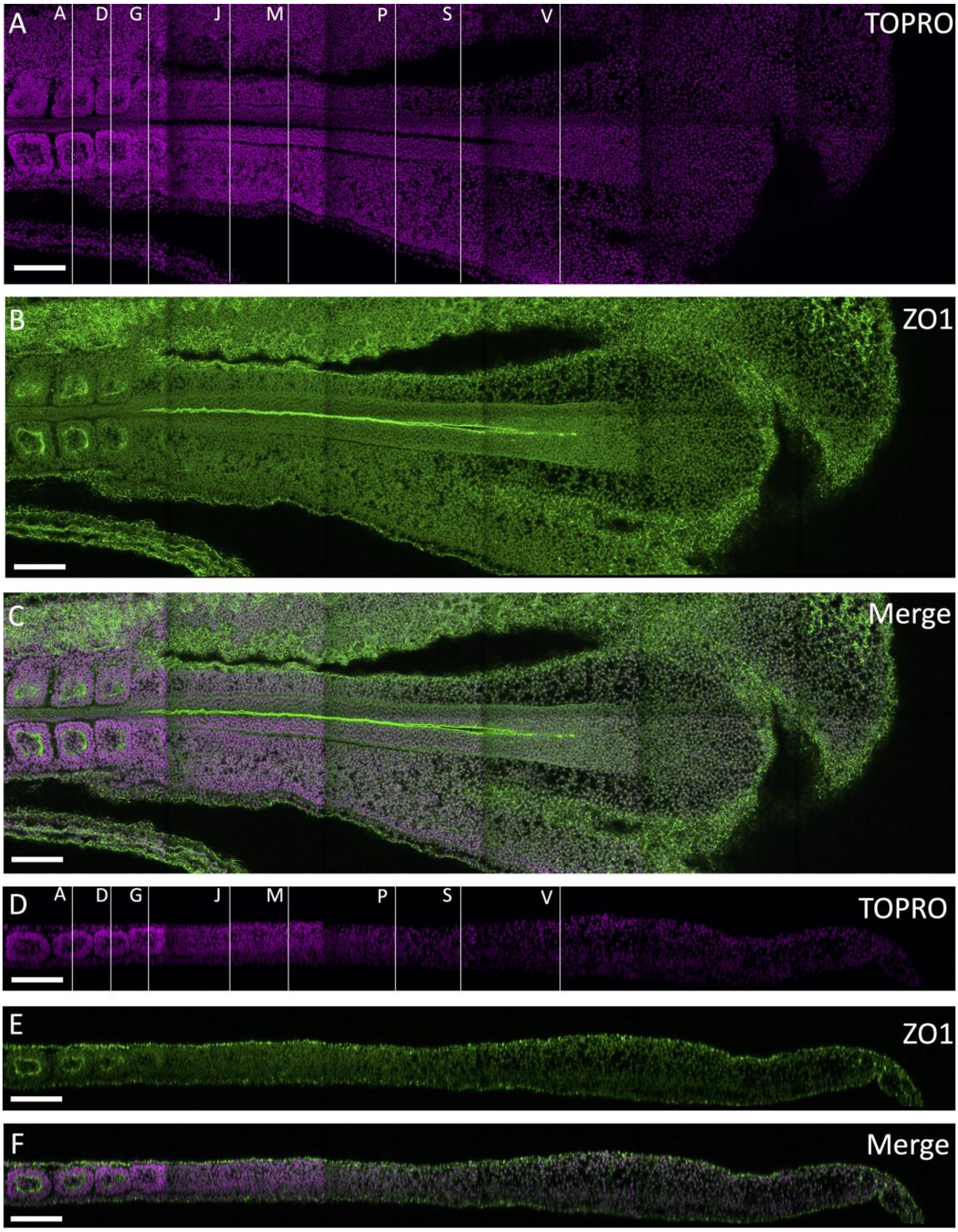

Fig S9. 3D localization of ZO1 and TOPRO, coronal and sagittal planes. A-C) Coronal planes (x,y). D-F) Sagittal planes (y,z). The white bars (A-V) in A, D correspond to the axial levels of transverse planes (x,z) presented in Fig S10. Scale bars are 100 μm. Anterior to the left.

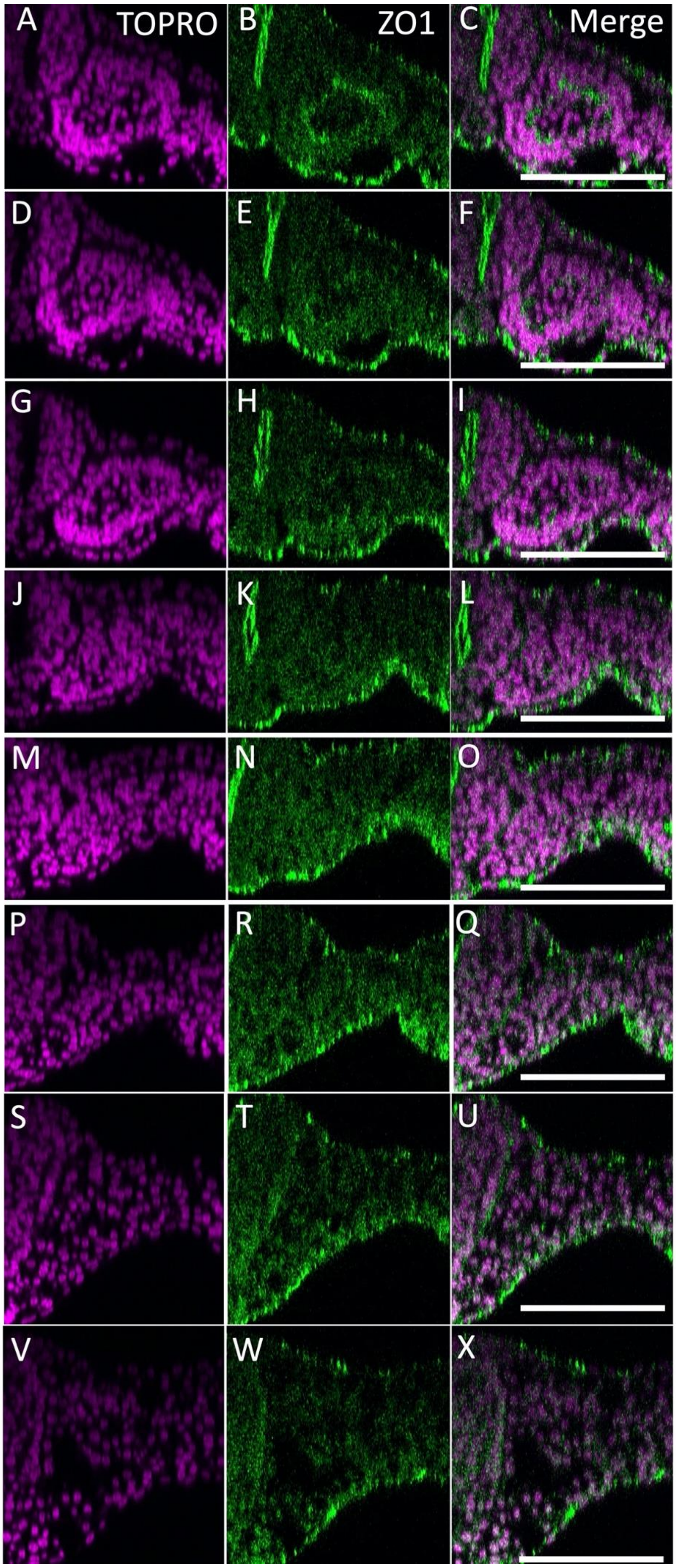

Fig S10. 3D localization of ZO1 and TOPRO, transverse planes. A-X) Transverse planes (x, z) at different axial levels as indicated in Fig S9 A, D (white bars). Dorsal to the top. Scale bars are 100μm.

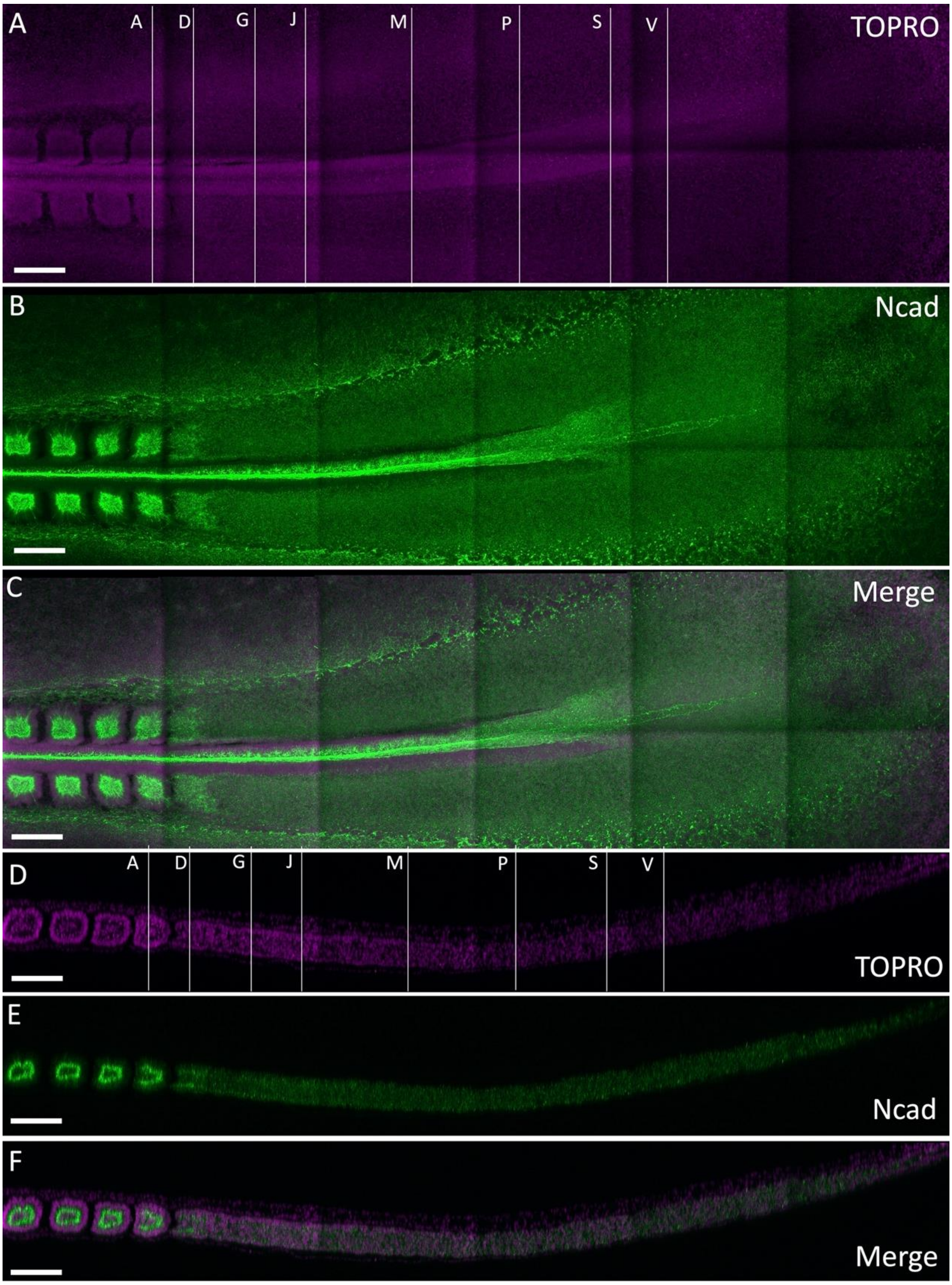

Fig S11. 3D localization of Ncad and TOPRO, coronal and sagittal planes. A-C) Coronal maximum intensity projections (x,y). D-F) Sagittal planes (y,z). The white bars (A-V) in A and D correspond to the axial levels of transverse planes (x,z) presented in Fig S12. Scale bars are 100 μm. Anterior to the left.

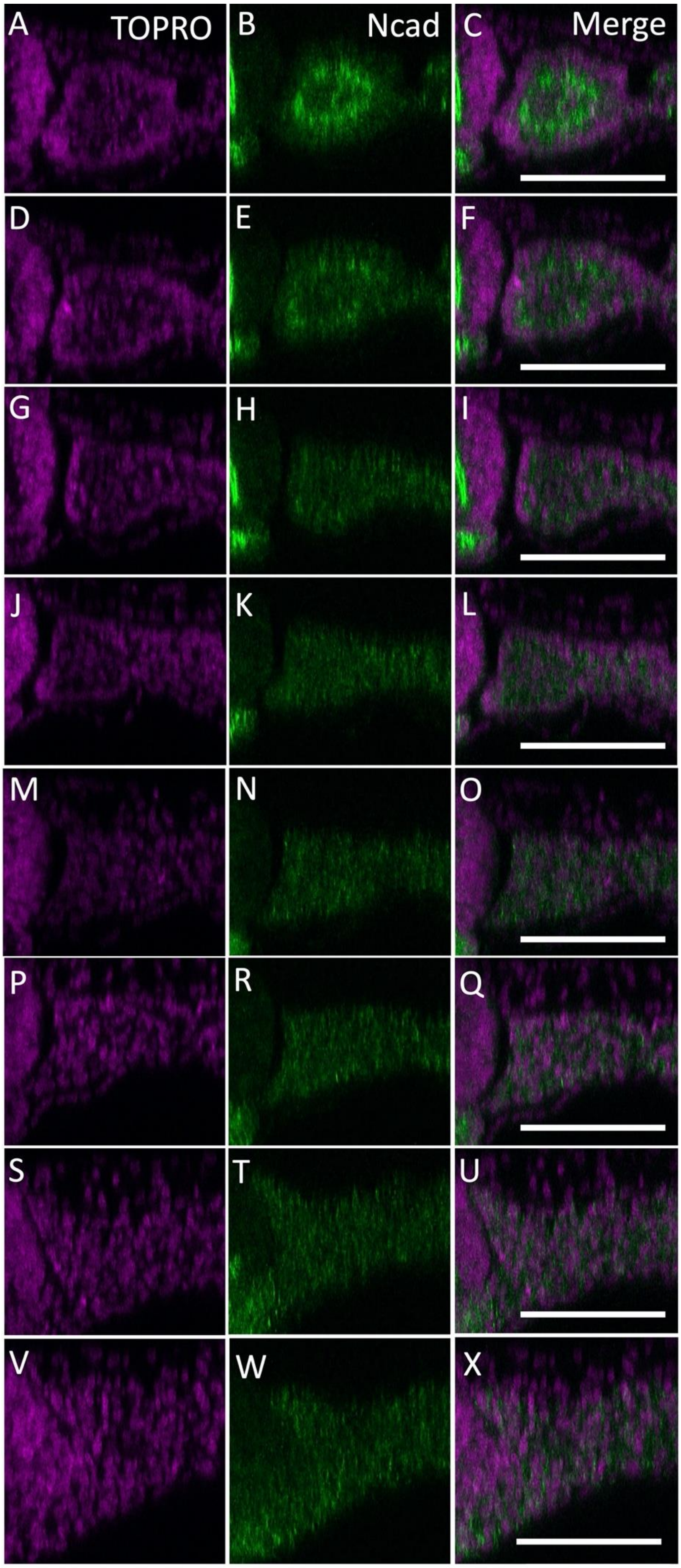

Fig S12. 3D localization of Ncad and TOPRO, transverse planes. A-X) Transverse planes (x,z) at different axial levels as indicated in Fig S11 A, D (white bars). Dorsal to the top. Scale bars are 100µm.

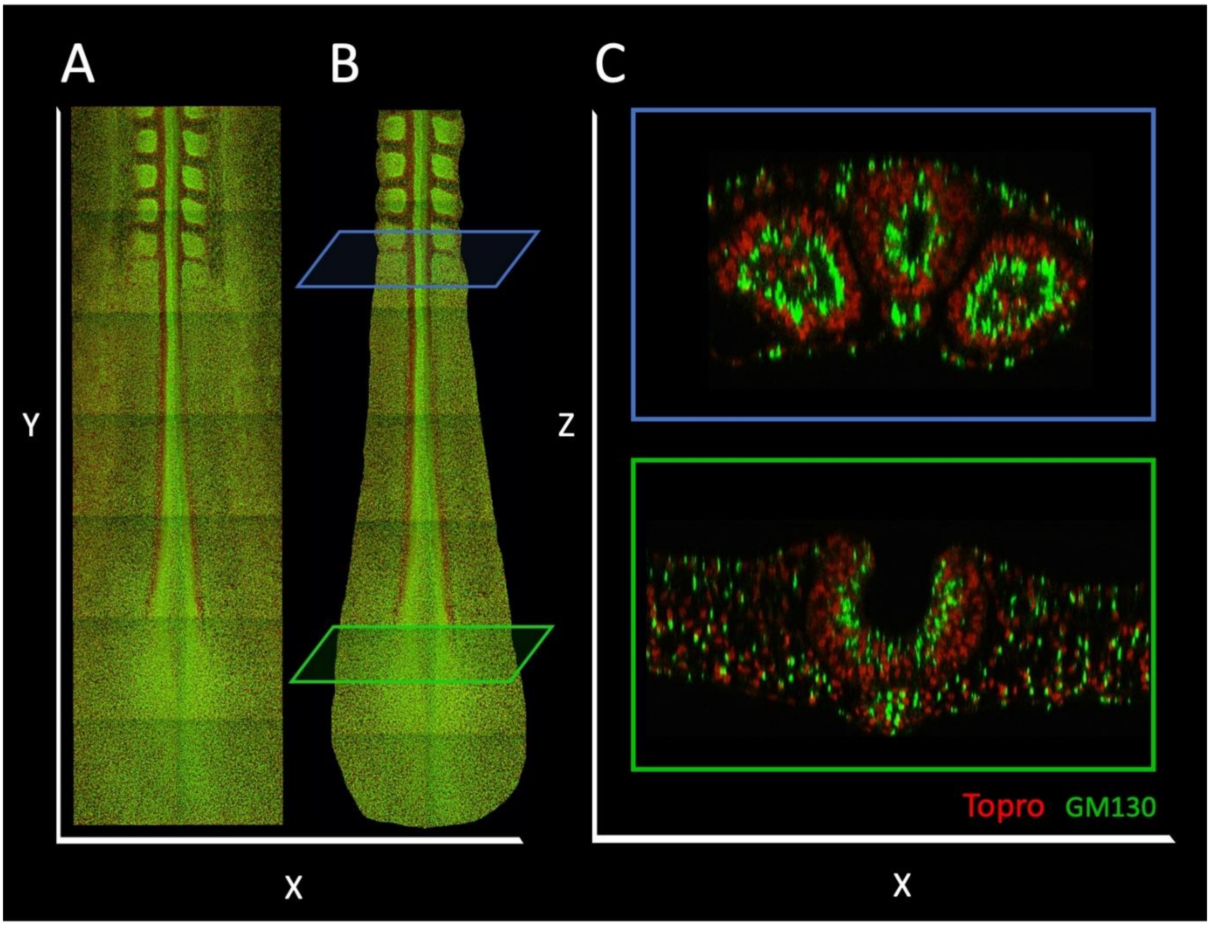

Fig. S13. Example of processing of 3D confocal images for analysis

A) Ventral view of maximum intensity projection of an embryo stained with TOPRO nuclear stain (Red) and for GM130, a Golgi ribbon marker (green). The image was stitched with 5% overlap. B) The same embryo with lateral and posterior tissues removed. C) X,Z view of the same embryo at the level of a formed somite (blue box) and posterior-PSM level (green box), at the planes indicated in B.

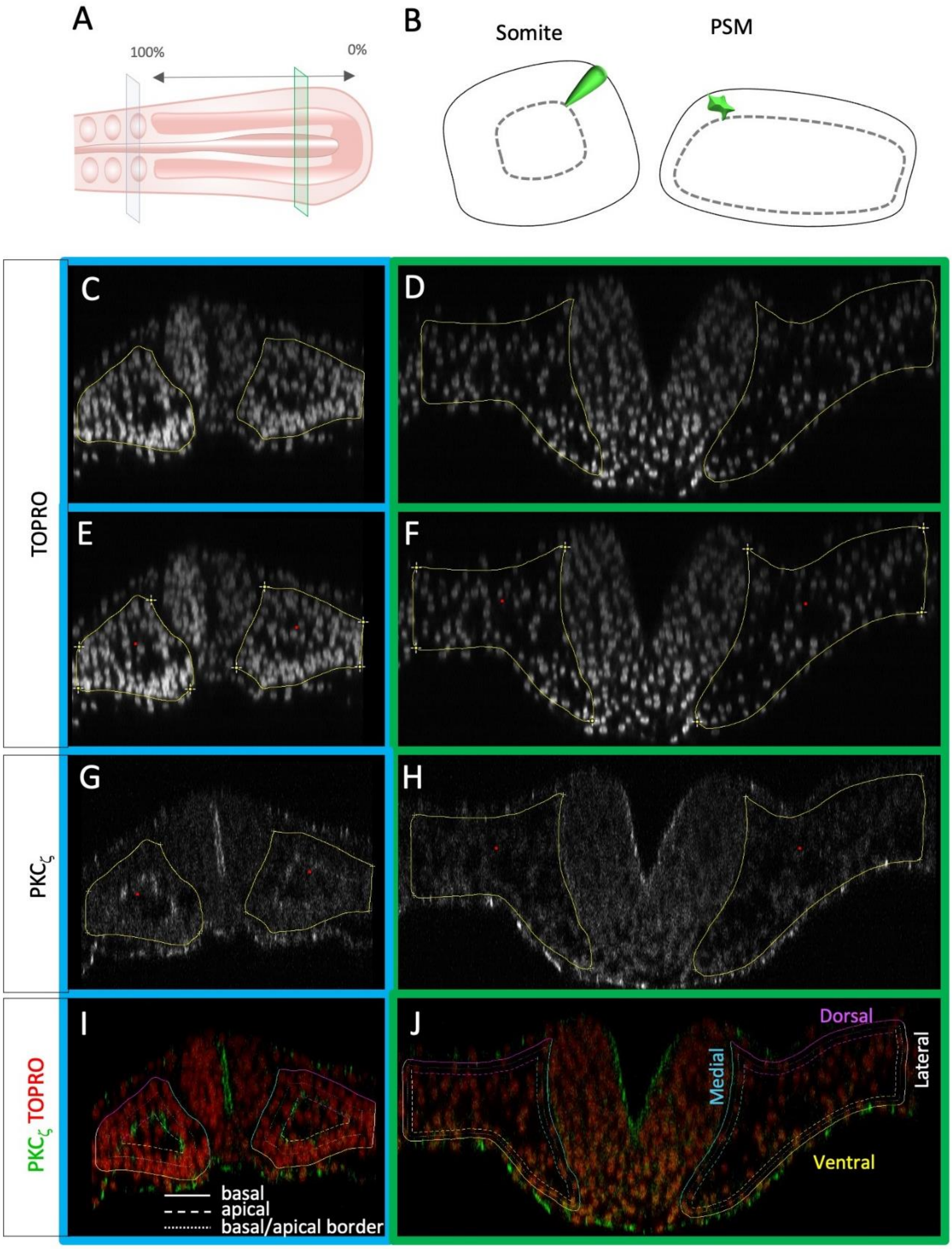

Fig. S14. Subdivision of somites and PSM into 4 domains, with apical-basal polarity revealed using PKCζ as apical marker and TOPRO nuclear stain. A) The length of the PSM was represented in % where the most posterior-PSM is 0% and the border between the newly formed somite and anterior-PSM is 100%. The somites fall above 100%. The blue box corresponds to somite

sections C, E, G, I, the green box represents posterior PSM (sections D, F, H, J). B) A given domain at a given PSM distance is an estimated length of a single cell. C-D) 3D images were resliced to obtain X, Z transverse optical sections. An outline (yellow line) was drawn on the TOPRO nuclear stain channel for somites (C) and the posterior-PSM (D). E-F) Four points (yellow crosses) were drawn manually and the centre of mass of cells automatically calculated in FIJI (red dots). Then straight lines from the yellow crosses to the centre of mass were virtually drawn to determine the borders between dorsal, ventral, medial and lateral domains of somites (E) and PSM (F). G-H) The outlines and the divisions of the domains were applied to the same embryo for the channel with a polarity marker i.e. PKCζ. I-J) TOPRO and PKCζ merged for the somites (I) and posterior-PSM (J) with the dorsal (magenta), ventral (yellow), medial (cyan) and lateral (white) domains halved to basal (solid line) and apical (dashed lines) zones.
